# Novel giardicidal compounds bearing proton pump inhibitor scaffold proceeding through triosephosphate isomerase inactivation

**DOI:** 10.1038/s41598-017-07612-y

**Published:** 2017-08-10

**Authors:** B. Hernández-Ochoa, G. Navarrete-Vázquez, C. Nava-Zuazo, A. Castillo-Villanueva, S. T. Méndez, A. Torres-Arroyo, S. Gómez-Manzo, J. Marcial-Quino, M. Ponce-Macotela, Y. Rufino-González, M. Martínez-Gordillo, G. Palencia-Hernández, N. Esturau-Escofet, E. Calderon-Jaimes, J. Oria-Hernández, H. Reyes-Vivas

**Affiliations:** 1 Laboratorio de Bioquímica-Genética, Instituto Nacional de Pediatría, Secretaría de Salud. Cd Mx., Mexico, 04530 Mexico; 2 Facultad de Farmacia, Universidad Autónoma de Morelos. Cuernavaca, Morelos, 62209 Mexico; 3 CONACyT-Instituto Nacional de Pediatría, Secretaría de Salud, Cd Mx., Mexico, 04530 Mexico; 4 Laboratorio de Parasitología Experimental, Instituto Nacional de Pediatría, Secretaría de Salud. Cd Mx., Mexico, 04530 Mexico; 5 Laboratorio de Neuroinmunología, Instituto Nacional de Neurología y Neurocirugía, Secretaría de Salud. Cd Mx., México, 14269 Mexico; 6 Instituto de Química, Universidad Nacional Autónoma de México, Circuito Exterior, Ciudad Universitaria. Cd Mx., Mexico, 04510 Mexico; 7 Laboratorio de Inmunoquímica, Hospital Infantil de México Federico Gómez, Secretaría de Salud. Cd Mx., Mexico, 06720 Mexico

## Abstract

Giardiasis is a worldwide parasitic disease that affects mainly children and immunosuppressed people. Side effects and the emergence of resistance over current used drugs make imperative looking for new antiparasitics through discovering of new biological targets and designing of novel drugs. Recently, it has determined that gastric proton-pump inhibitors (PPI) have anti-giardiasic activity. The glycolytic enzyme, triosephosphate isomerase (GlTIM), is one of its potential targets. Therefore, we employed the scaffold of PPI to design new compounds aimed to increase their antigiardial capacity by inactivating GlTIM. Here we demonstrated that two novel PPI-derivatives (**BHO2** and **BHO3)**, have better anti-giardiasic activity than omeprazole in concentrations around 120–130 µM, without cytotoxic effect on mammal cell cultures. The derivatives inactivated GlTIM through the chemical modification of Cys222 promoting local structural changes in the enzyme. Furthermore, derivatives forms adducts linked to Cys residues through a C-S bond. We demonstrated that PPI can be used as scaffolds to design better antiparasitic molecules; we also are proposing a molecular mechanism of reaction for these novel derivatives.

## Introduction

*Giardia lamblia* (*G. intestinalis, G. duodenalis*), the causal agent of giardiasis, is a flagellated protozoan parasite that lives in the small intestine. The infection can be asymptomatic or produce abdominal pain, chronic diarrhea^1, 2^, failure to thrive syndrome, and delays in the physical growth and cognitive-intellectual development of infants^1, 3, 4^. *Giardia* is a cosmopolitan parasite, but its prevalence is rising in developing countries; therefore, it is categorized into the neglected diseases group^5^. Besides, a rising incidence of giardiasis in children of daycare centers was identified, assigned it as a re-emerging infectious disease^6^. Giardiasis affects in all ages; however, immunosuppressed groups and children between 1–5 years old are mainly affected^7^. There are 280 million of cases worldwide reported with 500,000 new symptomatic cases every year^8, 9^. Current drug therapies include the use of benzimidazoles and 5-nitroimidazoles such as tinidazole, secnidazole, being metronidazole the most commonly employed. Nevertheless, there are reports describing drug resistance in strains from animals and human patients, as well as unwanted side effects by using such drugs^10–15^. The mentioned above, stand out the requirements of novel drug design therapies against this parasite, along with the discovery of new targets whose structural or functional changes disturb the viability and growth of parasite. It has been described the *Giardia* glycolysis as a potential biological target to design novel anti-giardiasic compounds^16, 17^. This metabolic pathway is considered one of the main sources of ATP synthesis for parasite^18^, since *Giardia* does not carry out oxidative phosphorylation^19^. The newly- synthetized Nitro(benzo)thiazole acetamide derivatives give rise to promissory results showing an enhanced *in vitro* anti-giardiasic activity. One of the potential drug targets is the glycolytic enzyme, fructose-1,6-biphosphate aldolase^20^. We showed that triosephosphate isomerase of *G. lamblia* (GlTIM), may be a potential specie-specific drug target. The enzyme is a homodimer that catalyzed the interconversion between D-glyceraldehyde 3-phosphate and dihydroxyacetone phosphate. We reported elsewhere that the chemical modification (derivatization) of the non-catalytic residue Cys222 by the gastric proton-pump inhibitor omeprazole, induce the total inactivation of GlTIM^16^. This Cys222 derivatization disturbs the hydrogen bond lattice that affects both the binding of substrate and the catalytic mechanism, similar to observed before with other compounds^21, 22^. Omeprazole also showed an *in vitro* anti-giardiasic effect that correlates with the GlTIM inactivation from cells, suggesting an *in vivo* involvement of this enzyme as target^16^.

**Figure 1:**
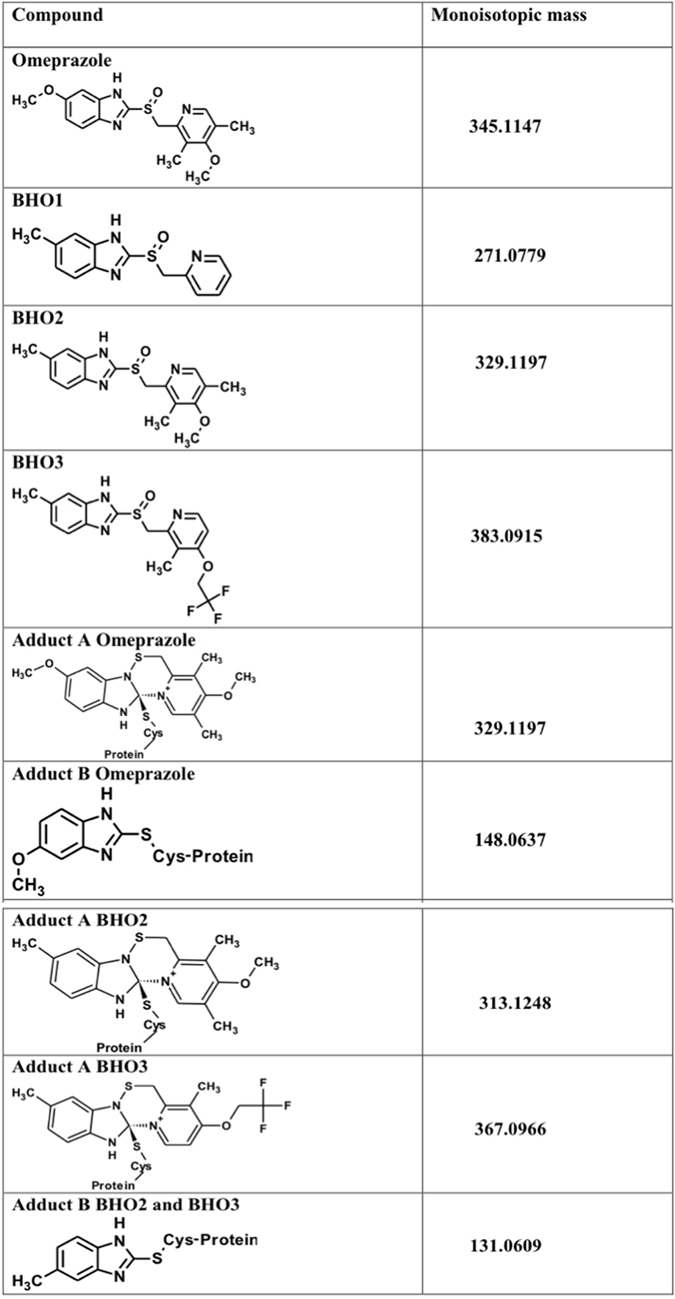
Structure and monoisotopic mass values of compounds and their adducts. The structures and masses were obtained by using ChemSketch softwarse (V14.01).

Here, we explore the properties that favor both the omeprazole reactivity to recombinant GlTIM and *in vitro* anti-giardiasic ability. To reach these aims, three derivatives named **BHO1**, **BHO2** and **BHO3** (Fig. 1), containing the scaffold of proton pump inhibitors were synthesized, purified and characterized. The derivatives have different substituents groups linked to the pyrimidine ring. The data showed that GlTIM inactivation is irreversible even when modified enzyme was treated with reducing agents, this suggests that covalent binding of compounds to Cys222 is not by disulfide bridge; nevertheless, the enzyme exhibited subtle structural disturbs. Data from mass spectrometry showed that the benzimidazole moiety of compounds is linked to Cys222, but also to Cys14 and C127. **BHO2** and **BHO3** showed better GlTIM inactivation capacity and *in vitro* anti-giardiasic effect than omeprazole, without cytotoxicity in mammalian culture cells. The data support a proposed mechanism of reaction that will let improve the reactivity of new drugs subsequently synthesized. As far as we know, this is the first study that performs a systematic evaluation of substituents to prove the GlTIM inactivation ability and their anti-giardiasic capacity.

## Results

### Design of proton-pump inhibitors derivatives

Recently, it has been reported that omeprazole exhibits antiprotozoan activity^16, 17, 23^ in which the glycolytic enzyme GlTIM, engage as potential drug target in the anti-giardiasic activity of this compound^16^. Continuing this line and to improve the interactions that increase the inactivation ability of GlTIM and consequently, raising the biological activity against trophozoites, we designed three derivatives named as **BHO1**, **BHO2** and **BHO3** based on the structure of common proton-pump inhibitor omeprazole, replacing the methoxy group by a methyl group at position 5 of the benzimidazole core and changing substituents in the pyridine ring as follows. **BHO1** without any substituents, to determine the influence of the alkoxy substituent at *para* position in the nucleophilicity and basicity of the pyridine ring. **BHO2** with pyridine ring substituted as omeprazole (3,5-dimethyl-5-methoxypyridine) and **BHO3** with the pyridine substituents of lansoprazole (3-methyl-4-trifluroethoxpyridine) (Fig. 1). The presence of alkoxy substituents is very important for the mechanism of action, because greater the nucleophilicity of the nitrogen atom of pyridine, the greater the formation of the spiro intermediate in Smiles rearrangement (activation) of prazoles.

**Figure 2:**
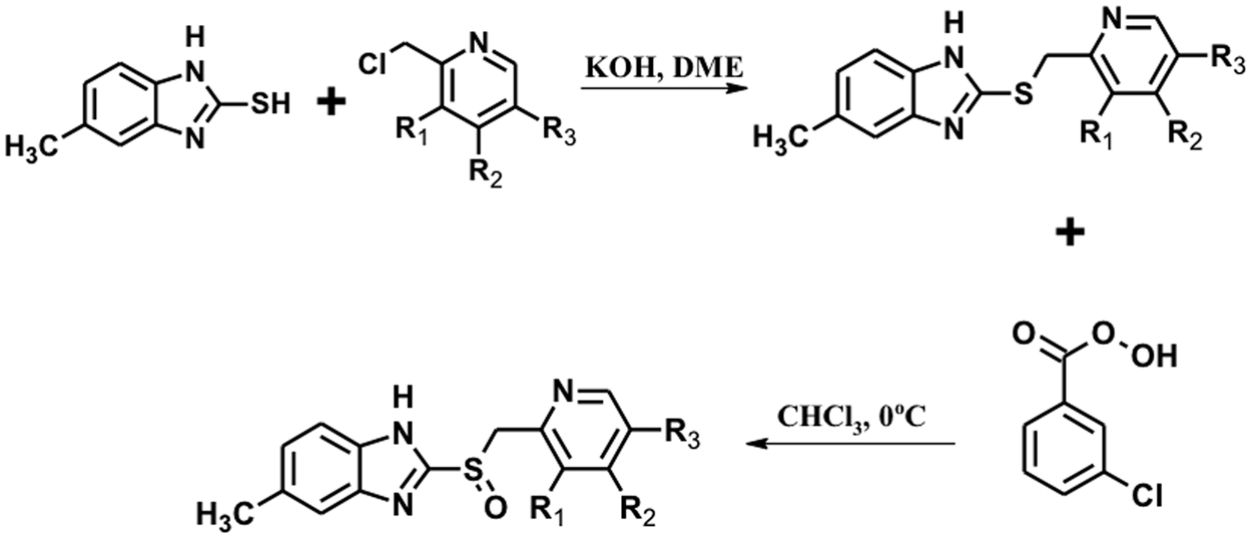
Synthesis of novel compounds. The structures were performed by using ChemSketch software (V14.01).

The derivatives were synthesized through two-steps method (Fig. 2). First, the 5-methyl-2-[(pyridin-2-ylmethyl)sulfanyl]-1*H*-benzimidazole precursors were prepared by nucleophilic substitutions between the 2-mercapto-5-methylbenzimidazole and one of the substituted 2-chloromethy pyridine; then the precursors were purified. Second, oxidation of thioether was done to obtain the sulfoxide products. Afterwards, the analogues were purified by silica gel column chromatography and their chemical structures confirmed by NMR spectroscopy (Figure S1 of Supplementary Material).

### *In vitro* screening of TIM inactivation

We previously reported that the target of several sulfhydryl compounds as well as omeprazole in GlTIM is the non-conservative residue Cys222^16, 21, 22^. This residue does not concern to the catalytic region, however, it is connected through hydrogen bond networks with both a zone accountable to bind the substrate and a region involved to the catalysis^24^. Hence, it is expected that derivatives affect the GlTIM activity through Cys222 derivatization but in a more efficient form. The C202A variant of GlTIM was selected for all experiments because of wild type enzyme has elevated propensity of generate oligomers of higher molecular weight. The oligomers are formed by linking dimers through disulfide bonds generated between two Cys202^25^. Substitution of Cys202 by Ala prevents multioligomerization leaving the enzyme only as dimer without functional or structural effects additionally observed^25^.

**Figure 3:**
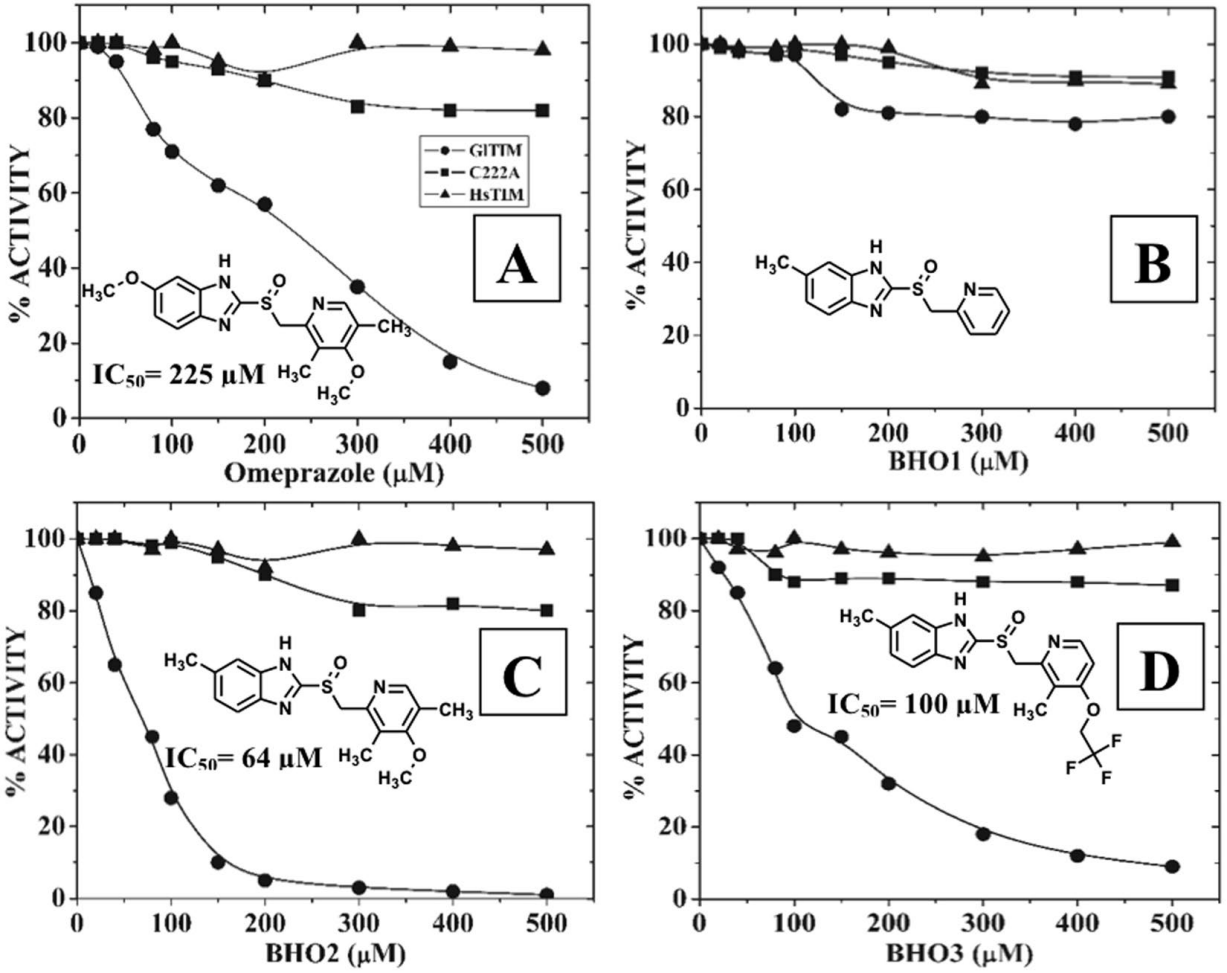
Inactivation assays of GlTIM with the compounds. GlTIM (200 µg/mL) was incubated with increasing concentrations of compounds. After 2 hours of incubation at 37 °C, the reactions were stopped by dilution and the remaining activity was determined as described in the material and methods section. The IC_50_ values from each compound are showed in each panel. The figures show representative experiments performed by triplicates.

We evaluated the effect of compounds incubating the enzyme with each one of them for 2 hours at 37 °C and the IC_50_ values were then calculated from the residual activity data. As reported before, omeprazole inactivated the GlTIM completely (Fig. 3A) showing an IC_50_ value of 225 µM. The IC_50_ values of **BHO2** and **BHO3** (Fig. 3C and D, respectively) were 64 and 100 µM, respectively showing stronger inactivation effect than omeprazole. In contrast, **BHO1** inactivated scarcely the enzyme (Fig. 3B).

The effect of omeprazole and analogues was also evaluated in mutant GlTIM C222A and human TIM (HsTIM), which contain an equivalent Cys residue, the Cys217 (Fig. 2 of Supplementary Material). As described before^16^, omeprazole affects marginally the activity of mutant GlTIM C222A while HsTIM was unaffected. In consonance with these results, **BHO2** and **BHO3** reduced the mutant GlTIM C222A activity only 20% using the higher assayed concentrations; derivatives did not induce inactivation in HsTIM (Fig. 3). Collectively, these data indicate that the structural target of derivatives is also the Cys222 likewise by perturbing the hydrogen bond networks linked to this residue, making to them specie-specific inactivators. Furthermore, the 20% inactivation at higher concentrations of derivatives in mutant C222A suggests some synergistic role for other Cys residues on the loss of enzyme activity. Indeed, it has been observed that derivatization of C222 along with other Cys residues like C14 by using 5,5′-dithio-bis(2-nitrobenzoic acid) (DTNB or Ellman’s reagent), promotes the GlTIM dissociation^21^.

To advance with the inactivating characterization of derivatives, we determined their second-order rate constant values of inactivation and then were compared with that obtained for omeprazole. **BHO1** was not more analyzed since it did not show important inactivation effect in GlTIM. As reported before, omeprazole inhibited the enzyme in an irreversible form^16^; similarly, **BHO2** and **BHO3** also inhibited to GlTIM irreversibly. The behavior of pseudo-first order rate constant values respect to the increasing concentrations of the three compounds was linear (Fig. 3 of Supplementary material). This conduct suggests that after a reversible complex between the enzyme and inhibitor is formed, it is rapidly transformed to a covalent complex. The second-order rate constant value of inactivation by omeprazole was 0.78 ± 0.016 M^−1^s^−1^, this value closely resemble to that of previously reported (i.e., 0.6 M^−1^s^−1^; 19); for **BHO2** and **BHO3**, the rate constant values were respectively 1.6 ± 0.1 and 1.2 ± 0.06 M^−1^s^−1^ showing that both analogs are more faster than the proton-pump inhibitor. Thus, the substitutions in both rings, the methoxy by methyl group in benzimidazole and the changes performed in pyridine, substantially improved the inactivating reactivity of analogues against GlTIM.

#### Reversibility assays of GlTIM inactivation

**Figure 4:**
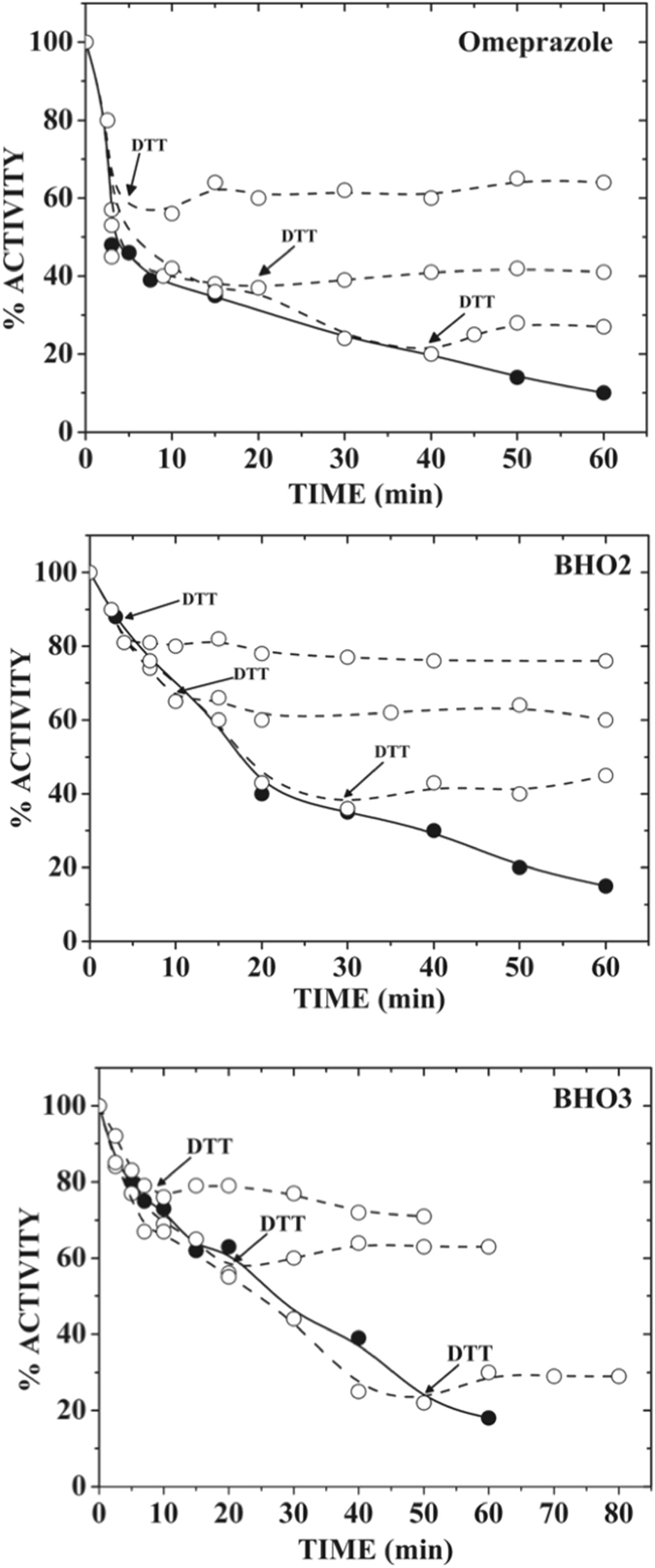
Failure of reactivation with reducing agent to derivatized GlTIM. GlTIM (200 µg/mL) was incubated with the different compounds (1 mM) at 37 °C; at various times, aliquots were withdraw and the reaction stopped by dilution, then the remaining activity was determined as described in the Material and Methods section. In parallel assays, the GlTIM was also incubated with the compounds, but at different times it was added 1 µL of DTT (5 mM final concentration) and the remaining activity monitored. The closed circles represent the control assay (i.e., without DTT treatment). Open circles represent the experiments with DTT treatment at different times. The arrows show the time in which DTT was added. The figures show representative experiments performed by triplicate.

To shed light about the reaction mechanism of omeprazole and derivatives in GlTIM, we performed experiments guided to determine if after its derivatization, the release of adducts attached to enzyme reverts the inactivation, assuming that binding is mediated by disulfide bridge. Hence, GlTIM was incubated at 37 °C with fixed concentrations of omeprazole, **BHO2** and **BHO3**; then aliquots were withdrawn in appropriated times in order to obtain around of 75, 50 or 20% of residual activity. Afterwards, we added to aliquots the reducing agent dithiothreitol (DTT) and their residual activities continued monitoring. Figure 4 shows that after DTT addition, the inactivating progression was arrested because of free compounds react with DTT instead of enzyme. However, regardless the residual activity in which stopped reaction, the enzyme was never reactivated for as long as the experiments lasted. These data indicate that the induced inactivation by anyone of compounds was irreversible. Such an effect may be explained considering that compounds do not bind with cysteine residues through disulfide bridges. DTT breaks the disulfide bridges by reduction and it does usually not disrupt another covalent bindings. Alternatively, irreversible inactivation may be also explained in terms of important structural perturbation of enzyme, which could not be reverted after releasing of the adducts.

We evaluated the first option by determining the residual free Cys after derivatization with compounds and afterwards reducing agent treatment. Thus, 6 mg/mL (214 µM) of GlTIM was incubated with or without each one of compounds (1 mM) during two hours at 37 °C. It is important to remark that at the protein concentration used for this aim, a considerable quantity was precipitated. Therefore, the incubation of the samples was arrested by centrifuging and then filtration through a 3 mL Sephadex G-25 column, and the number of free Cys quantified from the soluble protein. The GlTIM without treatment showed 3.95 ± 0.2 Cys/monomer; this was in concordance with the theoretical number of expected residues, i.e., 4 Cys/monomer. In contrast, the number of Cys per monomer obtained for omeprazole, **BHO2** and **BHO3** treatment was respectively 2.02 ± 0.02, 1.9 ± 0.2 and 2.15 ± 015. This indicates that two Cys residues were modified by treatment of compounds. An aliquot of each derivatized enzyme was withdrawn and incubated with 5 mM DTT during 15 minutes; then, the samples were filtered and the free Cys was again quantified. The number of Cys/monomer was 2.2 ± 0.2, 2,1 ± 0.22 and 1.8 ± 0.18 for omeprazole, **BHO2** and **BHO3**, respectively. Hence, the data showed that, notwithstanding the evaluated compound the number of free Cys per monomer does not change, showing that reducing agent did not be able of release the adduct from Cys residues. Therefore, the data strongly suggest that compounds are not attached to the Cys residues of GlTIM through disulfide bridges.

#### Structural changes of GlTIM by derivatization

**Figure 5:**
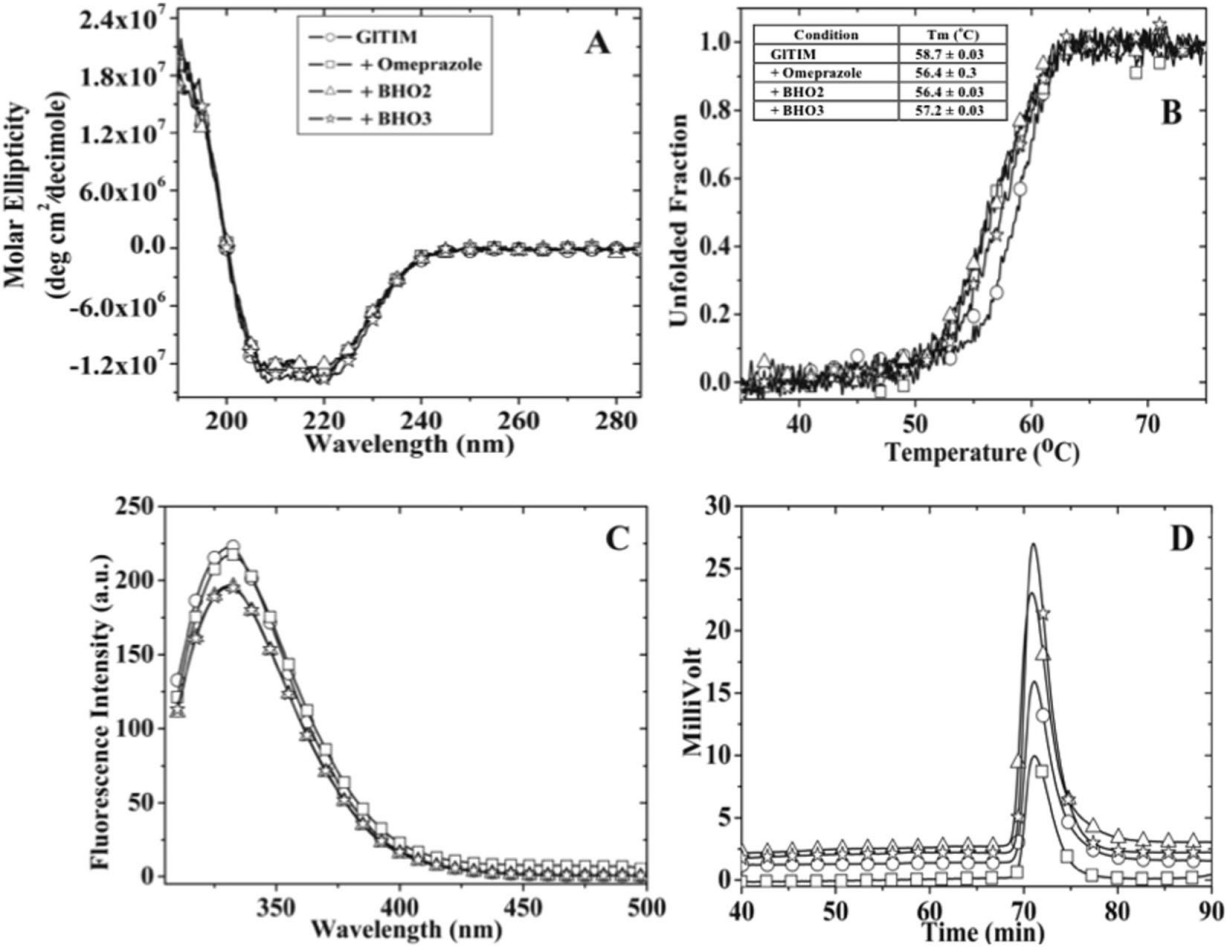
Structural studies of modified GlTIM with the Compounds. GlTIM (3 mg/mL) was incubated with the compounds (1 mM) during 2 hours at 37 °C; then the reaction was stopped by filtration and the structural experiments performed. For (**A**) CD spectra and (**B**) thermodenaturalization, the GlTIM concentration used was 200 µg/mL; the inset table contains the Tm values. For (**C**) Intrinsic fluorescence assays, the final enzyme concentration was 100 µg/mL. For size exclusion chromatography, the quantity of enzyme injected for each run was 20 µg (12 µL). Some symbols were removed from figures for a better clarity. The symbology used in panel A is the same for all panels.

Given that structural perturbation might contribute in the mechanism of irreversible inactivation, we evaluated the conformational changes of GlTIM by its derivatization with the compounds. We first investigated changes in the secondary structure by monitoring the circular dichroism (CD) signal of GlTIM modified by compounds and free of treatment; however, no appreciable changes were detected (Fig. 5A). Besides, the thermostability analysis following the CD signal at 222 nm showed subtle differences between the enzyme free of treatment and derivatized by compounds (Fig. 5B).

Changes in tertiary structure were evaluated by monitoring the intrinsic fluorescence properties of GlTIM. We found that derivatization induces a reduction in the maximum peak fluorescence value in which a 25% of signal was lost in the derivatized enzyme with **BHO3**, whereas close of 17% decreased by omeprazole and **BHO2** treatment. Otherwise, the mass center spectra of GlTIM were not affected by compounds treatment (Fig. 5C).

Finally, changes in quaternary structure were evaluated by using size exclusion chromatography. We did not find appreciable changes in the retention time between the enzyme without treatment and modified with different compounds (Fig. 5D). Collectively, the data suggest that chemical modification of GlTIM by omeprazole or derivatives induce local modifications to the structure, instead of global modifications.

#### Identification of adducts linked to Cys residues

To gain insight in the mechanism of GlTIM derivatization by compounds, we analyzed the modified enzyme with omeprazole, **BHO2** or **BHO3** by LC-MS/MS to identify the linked adducts in Cys residues. Thus, GlTIM was treated with omeprazole, **BHO2** or **BHO3** with the same procedure reported for Cys quantification.

The structures and monoisotopical masses of adducts were determined by considering the different mechanisms of omeprazole action previously reported (Fig. 1)^22–24, 26^. Moreover, since the derivatized Cys residues did not recover after the treatment with DTT, we assumed that all identified adducts were attached through covalent bonds that do not involve disulfide bridges. Table 1 summarized the modified peptides containing Cys residues derivatized.

**Table 1:** Identification of Adducts linked to Cys residues.

Peptide Sequences	Adduct (Δm)	Site	No. Spectra	Calculated Mass	Measured Mass	Error (ppm)
**OMEPRAZOLE**						
RPFIGGNFK**C**NGSLDFIK	148.06	Cys14	2	2160.09	2160.1	5.81
RPFIGGNFK**C** NGSLDFIK	148.06	Cys14	2	2161.07	2162.08	2.06
**C** NGSLDFIKSHVAAIAAHK	148.06	Cys14	3	2130.06	2131.12	7.36
ALEKGMTVIF**C**VG	148.06	Cys127	8	1515.81	1515.8	9.93
IIYGGSANGSN**C**EK	148.06	Cys222	1	1559.7	1560.72	3.68
IIYGGSANGSN**C**EKLG	148.06	Cys222	2	1730.79	1730.8	8.27
**BHO2**						
**C** NGSLDFIKSHVAAI	131.06	Cys14	1	1705.83	1706.84	0.9
PFIGGNFK**C** NGSLDFIK	131.06	Cys14	1	1988.95	1989	7.39
F**C**VGETLDERKANR	131.06	Cys127	2	1767.86	1768.86	0.62
IIYGGSANGSN**C**EK	131.06	Cys222	1	1542.7	1541.69	1.84
**BHO3**						
PFIGGNFK**C** NGSLDFIK	131.06	Cys14	1	1988.95	1991	0.27
**C** NGSLDFIK	367.09	Cys14	3	1362.59	1363.62	9.25
RPFIGGNFK**C**NGSLDFIKSHVAAIAAHK	367.09	Cys14	16	3364.7	3365.73	9.35
RPFIGGNFK**C** NGSLDFIKSHVAAIAAHK	367.09	Cys14	4	3365.69	3366.72	8.73
IIYGGSANGSN **C**EKL	131.06	Cys222	1	1656.76	1657.78	1.5
IIYGGSANGSN**C**EK	131.06	Cys222	2	1542.7	1541.69	0.84

The modified Cys appears in bold and underlined while the deamidated Asn residues appear underlined. The calculated monoisotopic mass values include the modifications and were calculated by using PeptideMass from ExPASy suite (http://web.expasy.org/peptide_mass/). The measured monoisotopic mass values were determined from LC-MS/MS data after their analysis with PeptideShaker suite. The error values correspond to the highest value obtained from all spectra for each peptide.

For omeprazole treatment, only the adduct including the benzimidazole moiety (Δm 148.06) contains fragmentation spectra of sufficient quality for a reliable assignment. This modification was identified in seven peptides containing Cys14, eight peptides enclosing Cys127 and another three peptides including Cys222 in their sequences. Similarly, for **BHO2** only the benzimidazole region of molecule was observed (Δm 131.06). Such a modification was identified in two peptides containing Cys14, two peptides containing Cys127 and one peptide with Cys222. **BHO3** treatment also rendered peptides with an adduct equivalent to benzimidazole moiety (Δm 131.06), one peptide containing Cys14 and three with Cys222. However, unexpectedly an adduct with Δm 367.09 was also clearly detected in 23 peptides containing Cys14, indicating that the reaction between this residue and **BHO3** render a product with both rings (see Fig. 1).

Altogether, the data indicate that compounds modify Cys222, but also the Cys14 and Cys127 are susceptible to derivatization. Besides, the route that follows the linkage of both omeprazole and **BHO2** induce the loss of pyridine ring. In contrast, **BHO3** is capable to form two adducts: the first one preserves both rings of molecule while the second one only conserves the benzimidazole ring like those generated by omeprazole and **BHO2**.

### Cytotoxic effect of compounds

**Figure 6:**
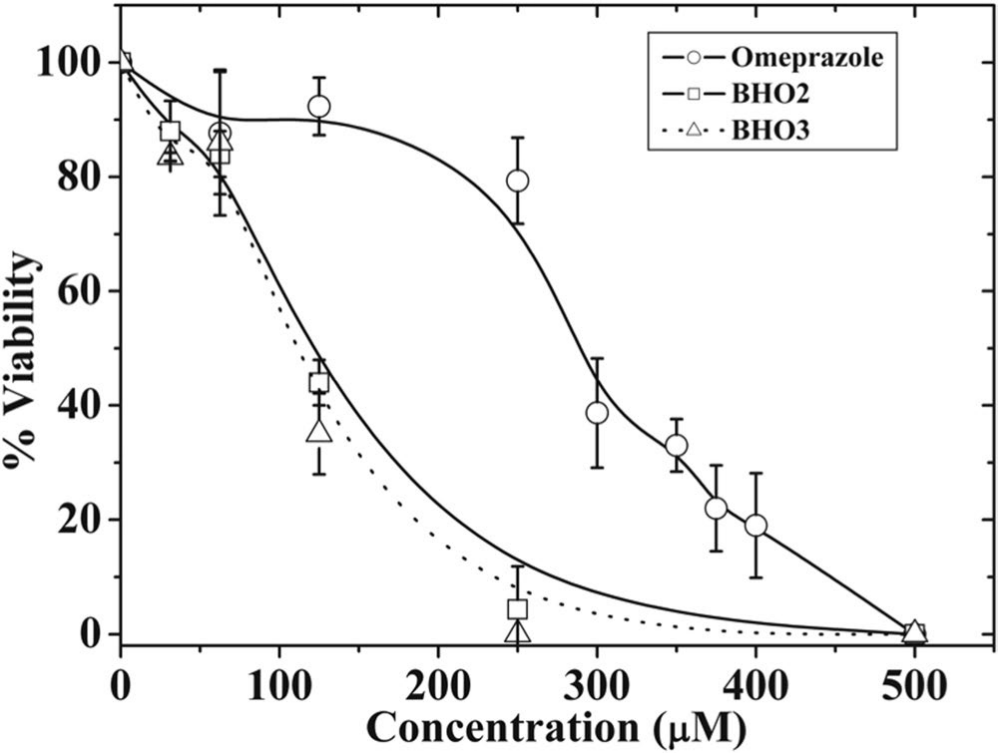
Anti-giardiasic activity of Compounds in trophozoites. Cultures of trophozoites (100,000 trophozoites/mL) were incubated during 48 hours at 37 °C with increasing concentrations of compounds. Afterward, samples were withdrawn and resuspended in a medium free of compounds. The samples were incubated for other 48 hours/37 °C and thereafter, the viability of trophozoites were quantified. The values represent ± standard deviation from three independent experiments.

The anti-giardiasic ability of compounds was tested in an *in vitro*. Figure 6 showed the viability percentage after incubation with rising concentrations of compounds. From these data, the IC_50_ value from each compound was calculated. Thus, for **BHO2** and **BHO3** the values (130 and 122 µM, respectively) were around of the half of that obtained for omeprazole (300 µM). These results indicate that, in parallel with the increased inactivator effect on GlTIM, changes in substituents increase the cytotoxic effect of analogues over the trophozoites.

The cytotoxic effect of these compounds was also evaluated in mammal cell cultures and their CC_50_ values calculated and depicted in Table 2. Omeprazole showed low cytotoxic effect over VERO cells displaying a CC_50_ of 14 mM, this value is 46-fold higher than that obtained for trophozoites cytotoxicity (see values inside parenthesis). For human fibroblasts, omeprazole did not show cytotoxic effect. Likewise, **BHO2** and **BHO3** exhibited CC_50_ values for mammal cells in the millimolar ranges, which are 38- to 61-fold higher respect to the IC_50_ obtained for trophozoites; thus, analogues also exhibited low *in vitro* intrinsic cytotoxicity over eukaryotic cells.

**Table 2:** Cytotoxicity test of compounds.

	Trophozoites	Human Fibroblasts	VERO cells
	IC_50_ (µM)	CC_50_ (µM)	CC_50_ (µM)
Omeprazole	300 ± 10.2	No effect	14,000 (**46**) [9,700–27,000]
BHO2	120 ± 2.6	5,000 (**38**) [4,144–6362]	8,000 (**61**) [5,338–21,515]
BHO3	122 ± 1.7	5,500 (**45**) [4,470–7,458]	4,700 (**38**) [3,566–7–245]

The IC_50_ values ± standard error from trophozoites were obtained from graphs depicted in Fig. 6. The CC_50_ values of mammal cells cultures were obtained as described under material and methods section. The numbers inside of parenthesis correspond to the ratio CC_50_/IC_50_ values between mammal cells and trophozoites. The numbers inside of squares parenthesis indicate de 95% confidence limits.

## Discussion

Owing to the rising of resistant strains of current drugs against *G. lamblia*, important efforts to search novel targets and design new anti-giardiasic drugs have been performed^20, 27, 28^. On this line, we and other research groups previously determined that omeprazole has anti-giardiasic activity^16, 17^. Further, it has been shown that the loss of trophozoites viability correlates with a reduction of endogen GlTIM activity, suggesting its participation as one of the drug targets^16^. Therefore, we consider to omeprazole as lead molecule that can be take it as scaffold to design better anti-giardiasic drugs. In consonance to this aim, we design three derivatives (**BHO1**, **BHO2** and **BHO3**) to increase their ability to inactivate the GlTIM, along with intensifying the giardicidal effect, but having low cytotoxic activities. The synthesis through two-steps method afforded yields above of 70%, where a later chromatographic step increased their purity (≥99%). Hence, excepting **BHO1**, the other two compounds improved the inactivation against GlTIM respect to omeprazole. The modest inactivating capability of **BHO1** (20%) along with the absence of substituents in its pyridine ring, disclose the essential contribution of these latter on the reaction mechanism. **BHO2** changed the methoxy by a methyl group and preserved the original substituents of omeprazole in the pyridine ring; this changes improved its solubility but notably, its reactivity against GlTIM (three-fold). For **BHO3**, nonetheless this compound had an increment of reactivity (two-fold), the contribution of trifluoroethoxy group in the pyridine ring was more limited. It must be underline that neither **BHO2** or **BHO3** increased their reactivity against mutant GlTIM C222A, confirming that GlTIM inactivation is promoted by derivatization of Cys222, as have been reported for other compounds^21, 22^.

The high specificity of reaction for the Cys222 of GlTIM compared with its equivalent in HsTIM, the Cys217, has been observed with several compounds other that omeprazole^21^. From latter studies and this one, it was concluded that at least two particularities may contribute to this specificity. The first is that pockets surrounding the Cys222 and Cys217 are quite different, i.e., from the seven residues forming the pockets only one is conserved in both enzymes. Consequently, the distribution of electric charges of each pocket is dissimilar. Furthermore, the accessible surface area value of the residues surrounding the Cys217 of HsTIM is higher than that in Cys222 from GlTIM^21^. Second, inactivation effects by C222 provoke perturbations of loop 7, a region whose interrelationship with loop 6 is essential for stability, ligand binding and catalysis of all TIM’s. In contrast, the Cys217 modification of HsTIM by compounds or mutations, did not affect appreciably its catalysis or stability^24^. This suggest that the pocket of Cys217 does not interact with some one of the mentioned secondary structures.

**Figure 7:**
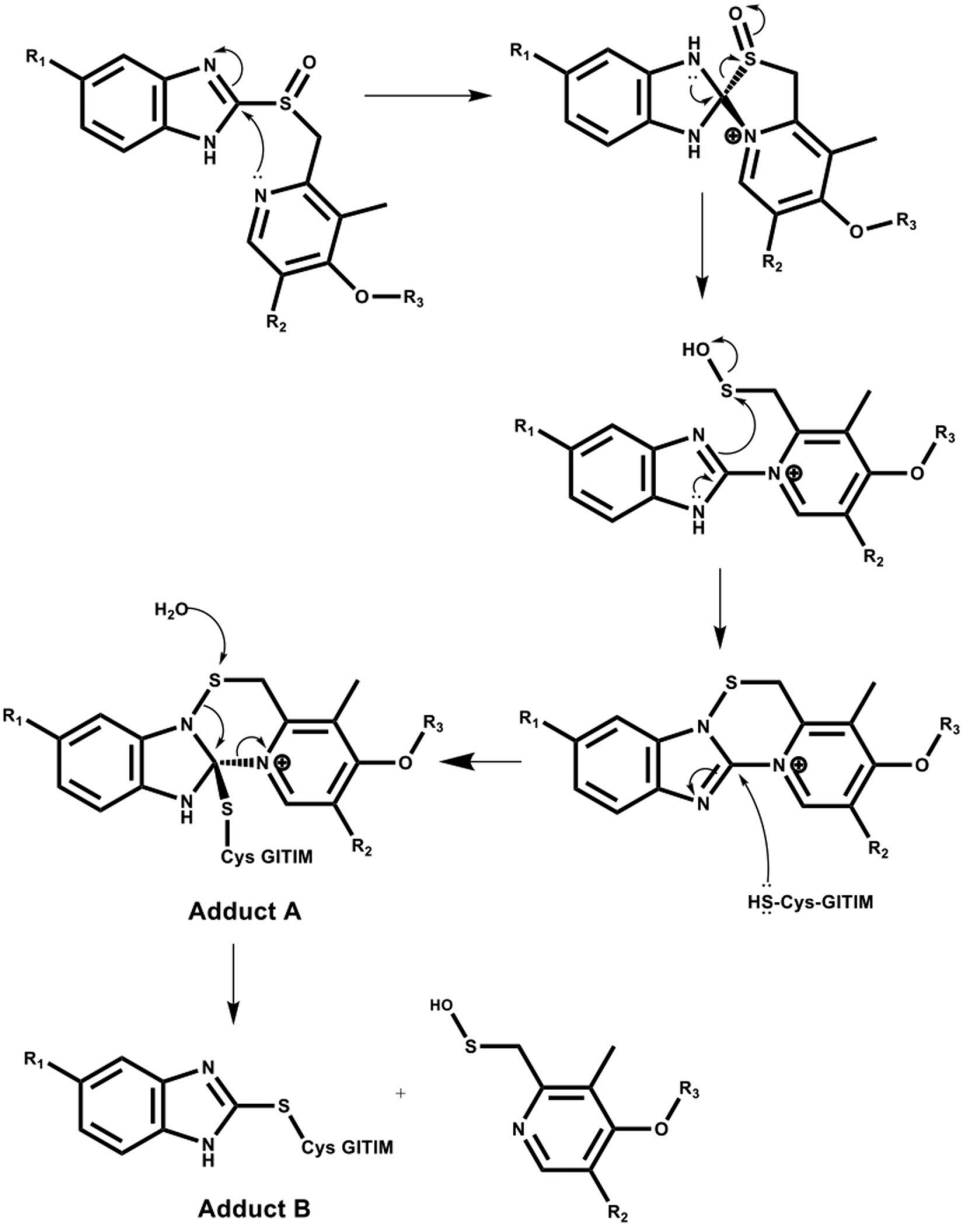
Proposed mechanism of formation of adducts (**A**) and (**B**). Mechanism of reaction of compounds and neutral pH.

Recently, it has been showed that derivatization with several proton-pump inhibitors pre-incubated in low pH, induces structural modifications in GlTIM and the generation of adducts attached by disulfide bridge that contain both rings;^23^ this suggests that reaction mechanism follows the route reported for proton-pump inactivation with omeprazole^29, 30^. In contrast, our data show that the compounds bind covalently to Cys residues by non-disulfide links; this data is of relevance to understand the chemical mechanism of reaction. In this regard, Im *et al*.^31^ proposed that at neutral pH, the benzimidazole moiety of omeprazole attached to a SH- group of mercaptanes by non-disulfide links. Given that our experiments were performed at neutral pH, it is reasonable to deduce that compounds bind to Cys yielding similar species. The data mentioned before, together with mass spectrometry results allow proposing a reaction mechanism of compounds (Fig. 7). The nitrogen of the pyridine ring acts as a nucleophile and uses its lone pair of electrons to form a bond to the electron deficient 2-carbon of the benzimidazole ring and form a spiro intermediate. By doing so, the aromatic character of the benzimidazole is lost and thus, there is a high tendency for this ring to re-aromatize. This can be achieved by a lone pair of electrons from nitrogen reforming the double bond and cleaving the S-C bond to form a sulphenic acid. The latter are highly reactive to nucleophiles and so a rapid reaction takes place involving an intramolecular attack by the NH group of the benzimidazole on the sulphenic acid to displace the hydroxyl group. A cationic tetracyclic pyridinium sulphenamide is formed, which acts as an irreversible enzyme inhibitor. It does so by forming a covalent bond to an accessible Cys residue through a nucleophilic substitution in which the -SH group of a Cys residue reacts with the C2 of benzimidazole ring, instead of the sulfur atom of sulphenamide, forming the adduct “**A**”. In particular, the reaction among Cys14 of GlTIM and **BHO3** concludes here generating this kind of adduct. On the other hand, omeprazole and **BHO2** render adducts only containing the benzimidazole ring. This circumstance may be explained considering the last part of reaction, in which a water molecule could attack the sulphenamide sulfur atom, breaking the S-N bond with the consequence displacing of pyridinium ring (which is a good leaving group), forming the adduct “**B**” (Fig. 7).

Derivatization of GlTIM at low enzyme concentration (7 µM) promotes barely structural modifications (Fig. 5); however, an increment of protein aggregation is obtained at concentrations beyond of 200 µM. This suggests that derivatization of GlTIM produces local perturbation of structure that generates solvent accessible hydrophobic patches, favoring its self-aggregation at high concentrations. Such propensity, together with an apparent heterogeneity of sample where concur enzyme populations containing different modified Cys, has precluded succeed in our efforts to crystallize the complex GlTIM-adduct at high resolution.

The *in vitro* studies showed that **BHO2** and **BHO3** have better anti-antigiardiasic effects than omeprazole, but without increasing their cytotoxicity in eukaryotic cells cultures. This data support our proposition about omeprazole can be used as scaffold to synthesize better anti-giardiasic compounds. Interestingly, albeit **BHO2** and **BHO3** displayed different inactivation rate constant values for recombinant GlTIM, they exhibited very similar IC_50_ values for the *in vitro* assays. These results suggest that besides of GlTIM, the compounds may have another target in *G. lamblia*. This could be a very interesting property since multi-target activity of drugs increases their anti-parasitic potential because of substantially decrease the probabilities that microorganisms generate resistance^32^. Additional studies are necessary to confirm such a proposal.

In conclusion, we demonstrate that gastric proton-pump inhibitors can be used as scaffold in drug-development to design better anti-parasitic molecules. Our data also offer a molecular mechanism of reaction in which the kind of adducts generated, together with the covalent bond that link to them with Cys residues have been clearly explained. Since the IC_50_ values for derivatives are still high, such information will be used to improve the reactivity of molecules synthesized in new refinement cycles and performing more medicinal chemistry studies. The ability of compounds to have multi-target activity in *G. lamblia* must be explored in order to identify their novel targets; such a characteristic appoint to these compounds as more-efficient anti-parasitic drugs.

## Material and Methods

### Reagents

Starting materials, Glyceraldehyde 3-phosphate (GAP), solvents, starting materials and other analytical grade reagents were purchased from Sigma-Aldrich, Merck, JT Baker and Amresco. The α-glycerol 3-Pi dehydrogenase and NADH were purchased from ROCHE. Calf serum and molecular biology reagents were supplied from Thermo Scientific, New England, Invitrogen and Applied Biosystems. Growth medium of trophozoites was purchased from DB and Sigma-Aldrich.

### Chemistry

Melting points were determined in an EZ-Melt MPA 120 automated melting point apparatus and the results are uncorrected. Reactions were monitored by thin layer chromatography using 0.2 mm precoated silica gel plates (60 F254; E. Merck). The ^1^H ^13^C and 2D NMR spectra were recorded in Bruker Avance 400 spectrometer (1H 400 MHz, 13C 100 MHz) and Bruker 700 Acend III spectrometer (1H 700 MHz, 13C 175 MHz). The chemical shifts are reported in ppm relative to the solvent resonance as the internal standard (CDC13; δH = 7.26 ppm; δC = 77.16 ppm; DMSO-d_6_: δH = 2.50 ppm, δC = 39.52 ppm). Mass spectrometry analysis was assayed on a JEOL JMS-700 spectrometer as previously showed^20^.

### General Synthesis of Compounds

Three new compounds were designed on the basis of the structure of the proton-pump inhibitors omeprazole and lansoprazole, maintaining the 2-(2-pyridylmethylsulfinyl)-1*H*-benzimidazole core. A methyl group at position 5 of benzimidazole ring replaced the methoxy group, the rest of substitutions were carried out on the pyridine ring with a common pattern: an alkoxy substituent at position 4 of pyridine ring, and a methyl group at position 3. Compounds were designed and synthesized in the Laboratorio de Química Farmacéutica, Universidad Autónoma del Estado de Morelos. We firstly prepared the precursors 5-methyl-2-[(pyridin-2-ylmethyl)sulfanyl]-1*H*-benzimidazole through of nucleophilic substitution (SN2) among the 5-methyl-1H-benzimidazole-2-thiol and a properly substituted 2-chloromethyl pyridine hydrochloride under a strong alkaline media. We monitored the total conversion of starting materials to pyridinsulfanylbenzimidazoles by TLC assays. After that, precursors were smooth oxidized with 3-chloroperoxybenzoic acid at 0 °C; this reaction was monitored every 30 minutes by TLC in order to prevent the superoxidation, until obtaining the total conversion of precursors to the corresponding sulfoxide product. Afterward, all analogues were purified by silica gel column chromatography; the analogues labeled as **BHO1**, **BHO2** and **BHO3** were stored at 4 °C until their use. Stock solutions of analogues were prepared by dissolving to them in DMSO or ethanol. Stock concentrations of **BHO1**, **BHO2** or **BHO3** were calculated from their absorbances at 299 nm under alkali conditions, using molar absorption coefficient (*ε M* ^−*1*^cm^−1^) values of 15476, 17304 and 13790, respectively. Omeprazole concentration was determined at 306 nm with *ε M* ^*−1*^cm^−1^ of 15474.

#### 5(6)-methyl-2-[(pyridin-2-ylmethyl)sulfinyl]−1*H*-benzimidazole (BHO1)

Light-brown solid. Yield 75%. Mp. 121.3–122.9 °C. ^1^H NMR (700 MHz, DMSO-d_6_) δ: 2.43 (3Hs, CH_3_), 4.69–4.76 (2H, AB system, *J* = 11.5 Hz CH_2_), 7.11 (1H, dd, *J* = 8.3, 1.0 Hz, H-6), 7.30 (1H, dt, *J* = 7.6, 1.0 Hz, H-3′ Py), 7.31 (1H, ddd, *J* = 7.6, 4.8, 1.0 Hz, H-5′ Py), 7.41 (1H, s, H-4), 7.53 (1H, d, *J* = 8.3 Hz, H-7), 7.75 (1H, td, *J* = 7.6, 1.8 Hz, H-4 Py), 8.51 (1H, ddd, *J* = 4.8, 1.8, 1.0 Hz, H-6′, Py) 13.31 (1H, broad s, NH) ppm. ^13^C NMR (175 MHz, DMSO-d_6_) δ: 21.3 (CH_3_); 61.9 (CH_2_); 123.2 (C-5′); 124.8 (broad, C-6), 125.3 (C-3′); 132.8 (broad, C-5); 136.9 (C-4′); 149.7 (C-6′); 150.7 (C-2′); 153.2 (C-2) ppm. Carbons C3a, C7a, C4 and C7 were not observed due to tautomeric equilibrium. MS calculated for C_14_H_13_N_3_OS: 271.0779, found: 271.0779.

#### 2-[(4-methoxy-3,5-dimethylpyridin-2-yl)methylsulfinyl]-5(6)-methyl-1*H*-benzimidazole (BHO2)

Light-brown solid. Yield 70%. Mp. 221.4–222.8 °C. ^1^H NMR (400 MHz, DMSO-d_6_) δ: 2.16 (3H, s, CH_3_), 2.19 (3H, s, CH_3_), 2.43 (3H, s, CH_3_), 3.68 (3H, s, CH_3_O), 4.68, 4.75 (2H, AB system, *J* = 13.5 Hz, CH_2_), 7.12 (1 H, dd, *J* = 8.3, 1.6 Hz, H-6), 7.42 (1H, s, H-4), 7.53 (1H, d, *J* = 8.3 Hz, H-7), 8.17 (1H, s, H-6′ Py), 13.4 (1H, broads, NH) ppm. ^13^C NMR (100 MHz, CDCl_3_) δ: 11.64 (CH_3_), 13.48 (CH_3_), 21.87 (CH_3_), 60.01(CH_3_O), 60.85 (CH_2_SO), 126.54 (C-5′), 127.15 (C-3′), 148.7 (C-2′), 149.83 (C-6′), 164.5 (C-4′); too broad resonances only detected through HMBC: 111.96 (C-4), 119.99 (C-7), 125.09 (C-6), 126.27, 133.1, 134.7, 142.18, 152.5 (C-2) ppm. MS calculated for C_17_H_19_N_3_O_2_S: 329.1198, found: 329.1197.

#### 5(6)-Methyl-2-({[3-methyl-4-(2,2,2-trifluoroethoxy)pyridin-2-yl]methyl}sulfinyl]-1*H*-benzimidazole (BHO3)

Light-brown solid. Yield 75%. Mp. 228.5–229.8 °C. ^1^H NMR (400 MHz, DMSO-d_6_) δ: 2.16 (3Hs, CH_3_); 2.44 (3Hs, CH_3_); 4.73, 4.81 (2H, AB system, J = 13.6 Hz, CH_2_-SO), 4.90 (2 H, q, J = 8.7 Hz, CH_2_-CF_3_), 7.09 (1H, d, *J* = 5.7 Hz, H-5′, Py), 7.12 (1H, broad, H-6), 7.48 (1H, broad, H-4), 7.57 (1H, broad, H-7), 8.28 (1H, d, *J* = 5.7 Hz, H-6′, Py), 13.42 (1H, broad s, NH) ppm. ^13^C NMR (100 MHz, CDCl_3_) δ: 11.13 (CH_3_), 21.86 (CH_3_), 60.92 (CH_2_SO), 65.71 (CH_2_O), 106.15 (C-5′), 123.54 (C-3′), 148.53 (C-6′), 150.69 (C-2′), 161.99 (C-4′); too broad resonance only detected through HMBC: 111.7 (C-4), 120.11 (C-7), 124.8 (C-6), 126.9, 134.45 ppm. MS calculated for C_17_H_16_F_3_N_3_O_2_S: 383.0915, found: 383.0915.

### Biological assays

#### Recombinant enzymes

For all assays we used mutant GlTIM C202A in order to work with the dimeric form of enzyme; this mutant was referred in the paper as GlTIM and was prepared as described before^24^. The recombinant GlTIM containing a His-tag in its NH-terminal together with a recognition region for tobacco each virus protease, was expressed in *Escherichia coli*. The GlTIM purification was performed by using IMAC method; after His-tag was removed^33^, the enzyme was stored at 4 °C in a buffer containing 100 mM triethanolamine, 10 mM EDTA, pH 7.4 (TE buffer). GlTIM purity was higher than 95% as determined by SDS-PAGE and MALDI-ToF mass spectrometry. Mutant of GlTIM C222A and human TIM (HsTIM) were prepared as described before^24, 33^. Protein content of GlTIM and HsTIM was calculated by using a *ε M* ^*−1*^cm^−1^ of 26,600 and 32,595, respectively. For all assays, the enzymes were freshly prepared with no more of two weeks of storage.

#### Activity assay

TIM activity was determined in a Cary50 spectrophotometer (Varian) at 25 °C using coupling assay following the conversion of D-glyceraldehyde 3-phosphate to dihydroxyacetone phosphate as described before^34^. The reaction mixture contained TE buffer (pH 7.4) supplemented with 0.2 mM NADH, 1 mM GAP, and 0.9 U of alpha-GDH. The reaction started by adding 5 ng/mL of TIM.

#### Inactivation assays

Omeprazole and compounds stock solutions were freshly prepared using 100% DMSO as solvent. For assays, TIM’s (0.2 mg/mL; 7 µM) were incubated in TE buffer (pH 7.4) at 37 °C with several concentrations of compounds; DMSO was maintained at 5% during incubation, this concentration did not affect the enzyme activity (data not shown). The reaction was stopped by diluting the enzyme 40,000 times in TE buffer and then, the remaining activity was determined. The second-order rate constants of inactivation were calculated from obtaining the pseudo-first order rate constant (*k*_*1*_) values at each fixed concentration of compounds by fitting them to an exponential decay. Then, the *k*_*1*_ values were re-plotted against the employed concentrations of each compound; the second-order rate constant value of inactivation was obtained from the slope of these plots^35^.

#### Spectroscopic and chromatographic characterization

To carry out the experiments, the derivatized GlTIM with different molecules was incubated with 2 mM DTT and then filtered through 3 mL Sephadex G-25 column to remove the unreacted compounds. All spectroscopic assays were carried out at protein concentration of 0.2 mg/mL (7 µM). Cysteine quantification was performed spectrophotometrically using de Ellman’s reagent as described before^22, 36^. Briefly, 200 µg of GlTIM were suspended in TE buffer supplemented with 5% SDS; after an incubation of 10 min at 25 °C, the enzyme was added to a cuvette containing 500 µL of TE buffer supplemented with 5% SDS and 1 mM DTNB. Cysteine quantification was done following the increase of absorbance at 412 nm, using an *ε M* ^*−1*^ *cm* ^*−1*^ of 13,600^36^.

Changes in secondary structure of proteins were monitored by circular dichroism (CD) in a Jasco-810 spectropolarimeter containing a thermostated Peltier-controlled cell holder. For CD experiments, the enzymes were previously equilibrated in 25 mM phosphate buffer, pH 7.4. Spectral scans from 190 to 280 nm at 0.1 nm intervals were performed at 25 °C using a quartz cell with 0.1 cm of path length. All CD data were reported as molar ellipticity using the expression described before^21^. Thermostability of modified GlTIM was evaluated recording the loss of CD signal at 222 nm during a temperature scan ranging from 25 to 90 °C and an increase of 1 °C/2.5 min. The unfolded fraction of enzyme and melting temperature (Tm) was determined as reported before^21^.

Changes of tertiary structure were analyzed following the protein intrinsic fluorescence in a Perkin-Elmer LS-55 spectrofluorometer. After proteins were excited at 280 nm, we recorded the emission spectra from 310 to 500 nm with a scan speed of 150 nm/min and using excitation and emission slits of 5 and 4 nm, respectively. We used phosphate buffer without protein as blank spectra and was subtracted from all fluorescence data. Changes in quaternary structure were evaluated by size exclusion chromatography (SEC) employing a Perkin-Elmer series 200 HPLC and a silica gel base column (YMC-Pack Diol-120) previously equilibrated with a mobile phase containing 150 mM NaCl, 50 mM potassium phosphate, pH 7.0. The system was calibrated using a gel filtration standard kit (Bio-Rad). Flow was maintained at 0.1 mL/min, the elution of proteins was monitored by their absorbance at 280 nm.

#### Identification of adducts bound to GlTIM through Liquid Chromatography coupled to tandem mass spectrometry (LC-MS/MS)

GlTIM (6 mg/mL; 214 µM) was incubated with 1 mM of compounds (omeprazole, **BHO2** and **BHO3**) during 2 hours in TE buffer at 37 °C. Then the samples were incubated with 2 mM of DTT during 10 min at 25 °C; the samples were later centrifuged at 15,000 × *g*/15 min at 25 °C and supernatant filtrated by 3 mL Sephadex G-25 column equilibrated with Milli-Q water. After that, the samples were totally desiccated and sending to the CBC-UIC Research Resource Center Mass spectrometry, Metabolomics and Proteomic Facility (University of Chicago, Illinois) for performing an LC-MS/MS analysis. The proteins were digested by trypsin and the resulting peptides were applied to a Dionex Ultimate 3000 HPLC system coupled to a Thermo Fisher Scientific Orbitrap mass spectrometer. The obtained data were analyzed by using MAXQUAT Software.

We performed an additional in-house strategy of analysis by using the Peptide Shaker suite (V 1.12.2)^37^. To this purpose, the raw data were transformed to Mascot generic format files for the analysis; a target-decoy database was built including mutant GlTIM-C202A and 116 sequences reported as potential proteins contaminants. Three*.mgf* data containing spectra data of modified GlTIM with omeprazole, **BHO2** or **BHO3** were submitted to the bundled SearchGUI tool (V3.0.3). We added five new post-translational modifications belonging to the compounds (Fig. 1). For omeprazole, adducts **“A”** (C_17_H_18_N_3_O_2_S, Δm 329.1197) and **“B”** (C_8_H_8_N_2_O, Δm 148.0637) were added. For **BHO2** and **BHO3**, their corresponding adducts **“A”** (C_17_H_18_N_3_OS, Δm 313.1248 and C_17_H_15_F_3_N_3_OS, Δm 367.0966, respectively) were added. Finally, only one adduct **“B”** was added since **BHO2** and **BHO3** generate the same one (C_8_H_7_N_2_, Δm 131.0609). In addition, the variable modifications of methionine oxidation (Δm 15.9949), and asparagine deamidation (Δm 0.9840) were also included. The m/z tolerance values of the precursors and fragments were 10 ppm and 0.9 Da, respectively. From suite, X!Tandem version 2015.12.15^38^, MyriMatch^39^, MS-GF + ^40^, OMSSA^41^ and Comet^42^ were used as search engines. Modifications identified were confirmed by manual analysis by using MS-Product bundled in Protein Prospector Suite (V5.17.1; http://prospector.ucsf.edu/prospector/mshome.htm).

#### Susceptibility assays of *Giardia* trophozoites

We used the *G. lamblia* WB reference isolate in all experiments. The splitting method used to our susceptibility assays is described below. Cultures with 100,000 trophozoites/mL on TYI-S-33 medium were incubated during 48 hours at 37 °C supplemented with increasing concentrations of compounds ranging from 32.5 to 500 µM. Afterward, samples were chilled in ice for 15 minutes, washed with medium and resuspended in 50 µL of medium free of compounds and antibiotic. Then, trophozoites were diluted in 1 mL of medium free of compounds and antibiotic, incubated for 48 hours at 37 °C and then, the viability of trophozoites was manually quantified by using a Neubauer chamber; the viability percent and IC_50_ was subsequently calculated.

#### Cytotoxicity assays in eukaryotic cells

We used the cell lines VERO (ATCC:CCL-81) and gingival human fibroblasts (kindly provided by Dra. María Dolores Farfán from Facultad de Odontología, Universidad Nacional Autónoma de México). The assay procedure is described below. Cells were maintained in a DMEM medium supplemented with antibiotic-antimycotic (1X), L-glutamine (1X) and 10% fetal bovine serum. Cells were incubated in 25 mL plastic culture flasks (Nunc) at 37 °C and 5% CO_2_. After the cell cultures grew to confluence, we determined the cell viability by using Trypan Blue method; then, cells were seeded at 4 × 10^4^ cells/well in 100 µL culture medium during 24 hours. Afterwards, the compounds were added in a volume of 100 µL using serial dilutions ranging from 500 µM to 31.2 µM; then, the cultures were incubated during 24 hours. DMSO was maintained constant at 0.2%; a control was performed by incubating cells with this solvent concentration but in absence of compounds. After incubation time, we measured the cell viability by using MTT (3-(4,5-Dimethyl-2-thiazolyl)-2,5-diphenyl-2H-tetrazolium bromide; Roche Diagnostics) method. Briefly, 10 µL MTT (5 mg/mL) was added to cell cultures and incubated at 37 °C during four hours; then, 100 µL of solution containing 10% SDS and 0.01N HCl was added. Thereafter, the formazan products were spectrophotometrically quantified at 570 nm (Labxystem Uniskan, Manchester). The CC_50_ (median cytotoxicity concentration) and CI_95_ (95% confidence interval) values from each experiment were determined from the concentration-percentage plots of cell viability obtained by logistic regression. Statistical analysis was performed using the SPPSS software (version 17.0).

## Electronic supplementary material

Supplementary material
