# Supplementary material for: Novel giardicidal compounds bearing proton pump inhibitor scaffold proceeding through triosephosphate isomerase inactivation
Source: Sci Rep. 2017 Aug 10;7:7810. doi: 10.1038/s41598-017-07612-y (PMC5552691; doi:10.1038/s41598-017-07612-y)

SUPPLEMENTARY MATERIAL

Novel Giardicidal compounds Bearing proton pump inhibitor Scaffold proceeding through Triosephosphate isomerase inactivation

Hernández-Ochoa B[1]., Navarrete-Vázquez G[2]., Nava-Zuazo C[2]., Castillo-Villanueva A[1]., Méndez ST[1], Torres-Arroyo A[1]., Gómez-Manzo S[1]., Marcial-Quino J[3]., Ponce-Macotela M[4]., Rufino-González Y[4]., Martínez-Gordillo M[4]., Palencia-Hernández G[5]., Esturau-Escofet N[6]., Calderon-Jaimes E[7]., Oria-Hernández J[1*]., Reyes-Vivas H[1*].

S1. Nuclear Magnetic Resonance Spectra of derivatives.

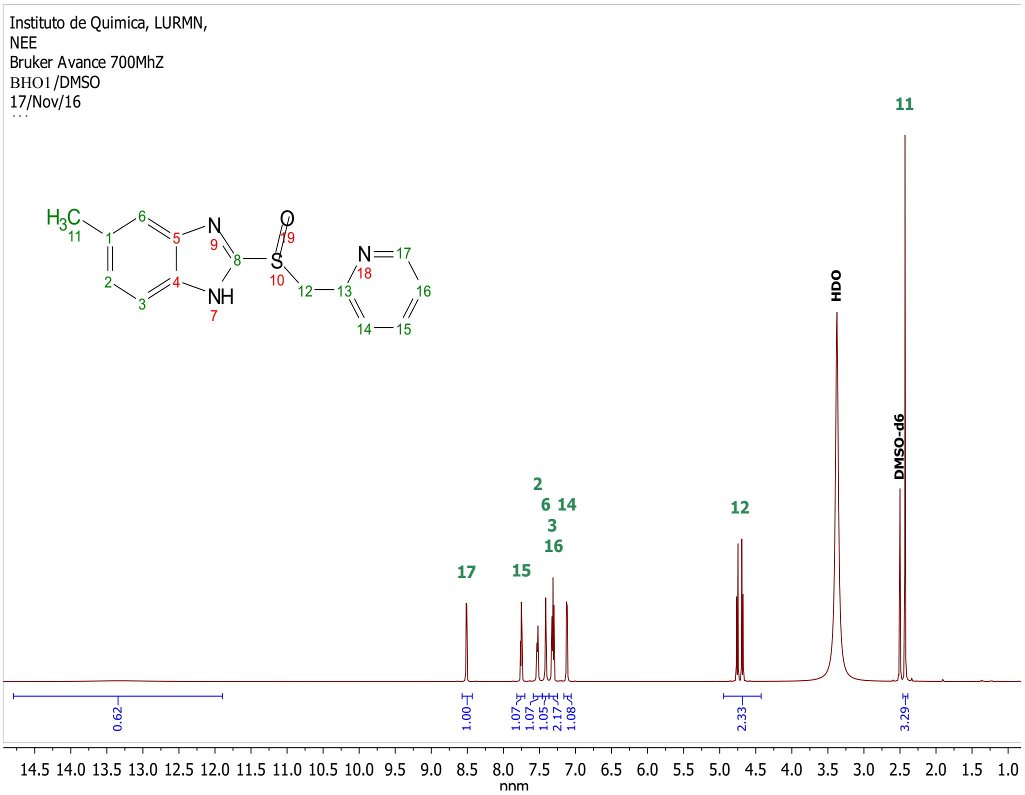

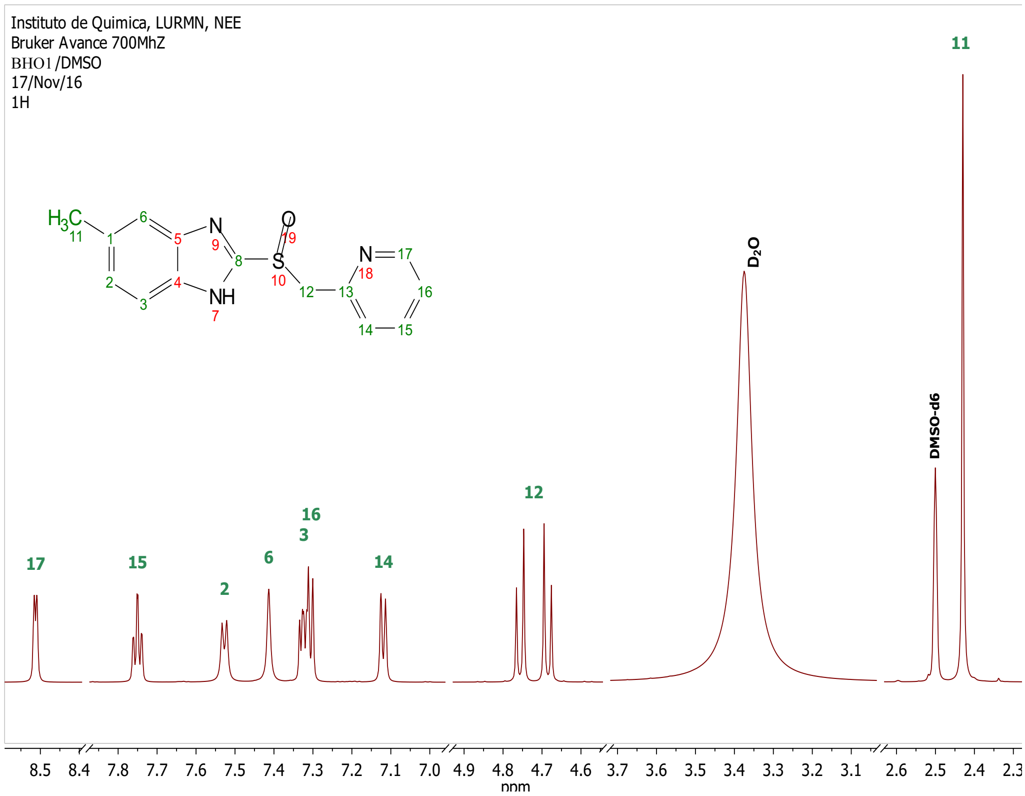

Instituto de Quimica, LURMN, NEE
Bruker Avance 700MhZ
BHO1/DMSO
17/Nov/16
1H
D2O
DMSO-d6
ppm

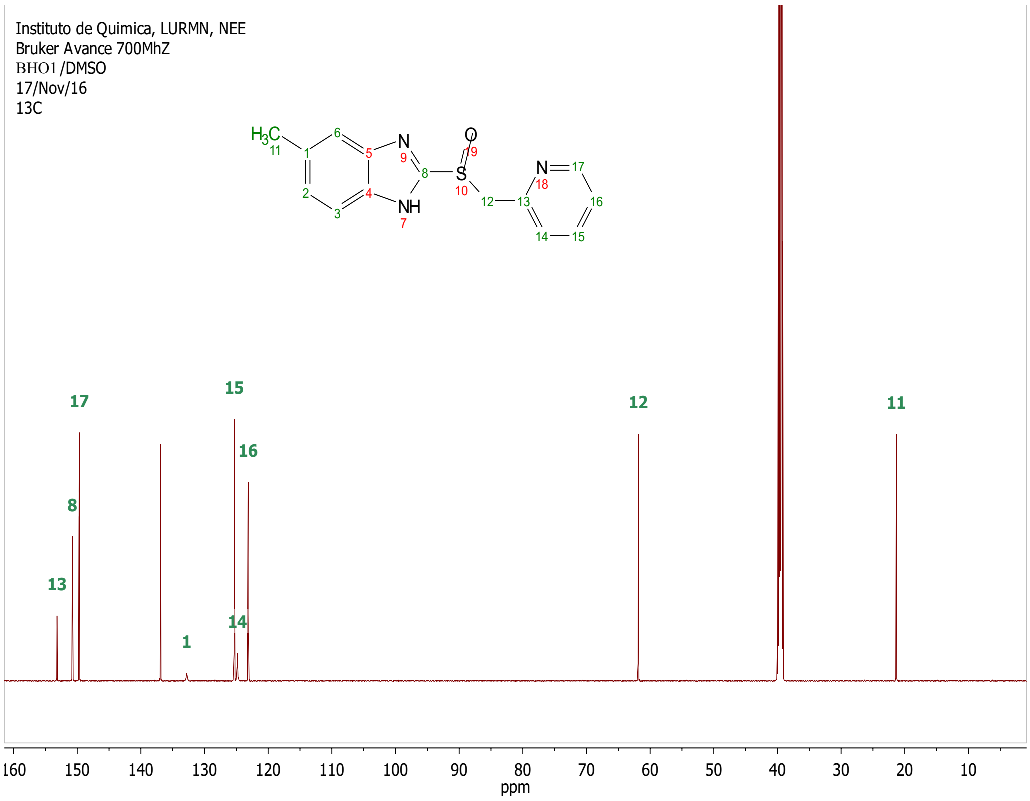

Instituto de Quimica, LURMN, NEE
Bruker Avance 700MhZ
BHO1/DMSO
17/Nov/16
13C
ppm

Instituto de Quimica, LURMN, NEE
Bruker Avance 700MhZ
BHO1 /DMSO
17/Nov/16
COSY

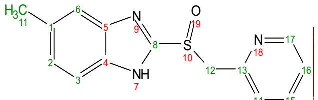

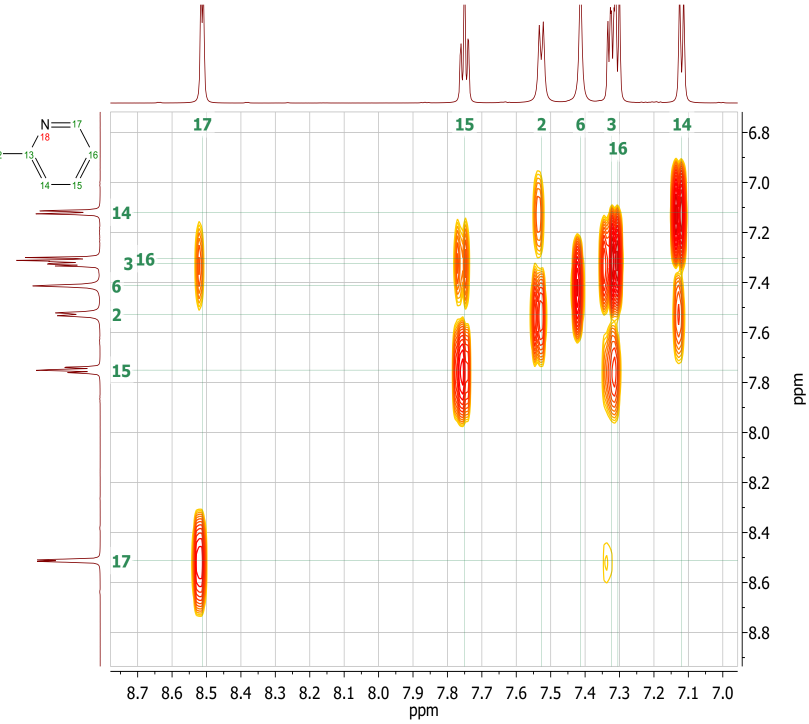

Instituto de Quimica, LURMN, NEE
Bruker Avance 700MhZ
BHO1 /DMSO
17/Nov/16
HMBC

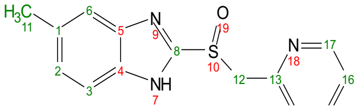

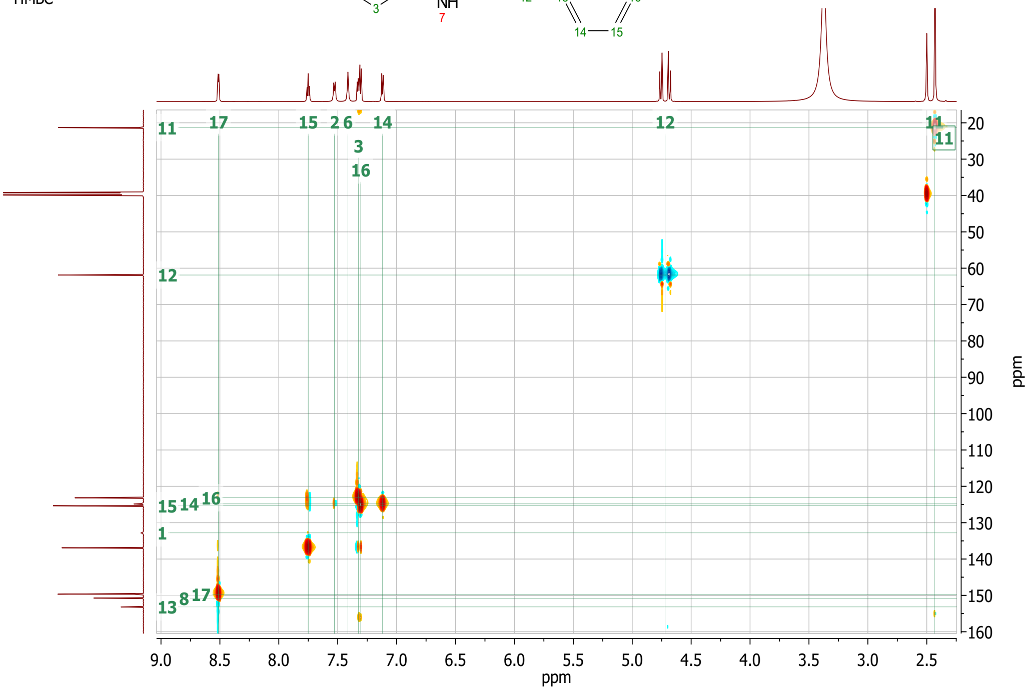

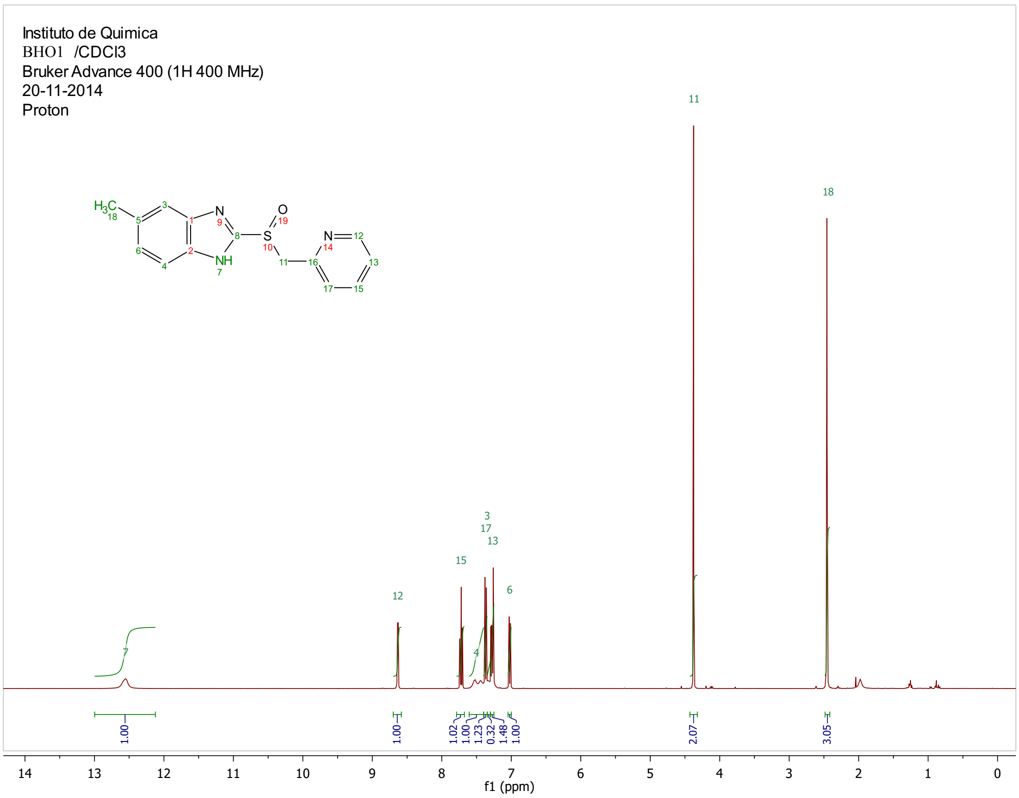
Instituto de Quimica
BHO1 /CDCl3
Bruker Advance 400 (1H 400 MHz)
20-11-2014
Proton
f1 (ppm)

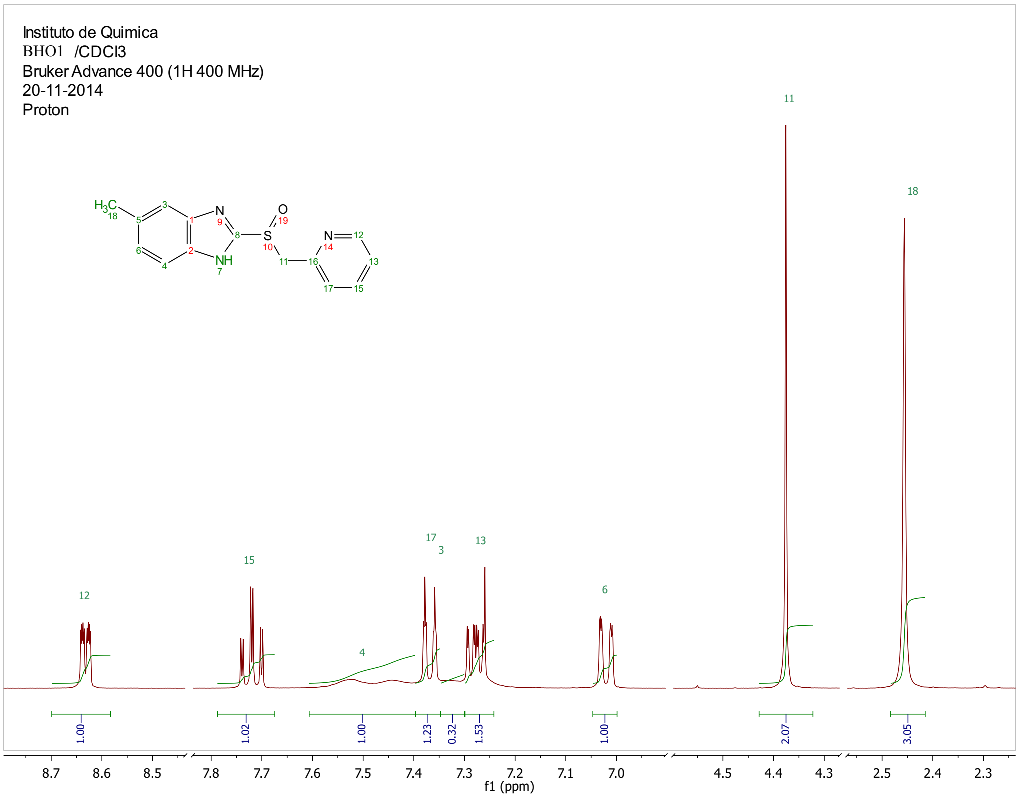
Instituto de Quimica
BHO1 /CDCl3
Bruker Advance 400 (1H 400 MHz)
20-11-2014
Proton
f1 (ppm)

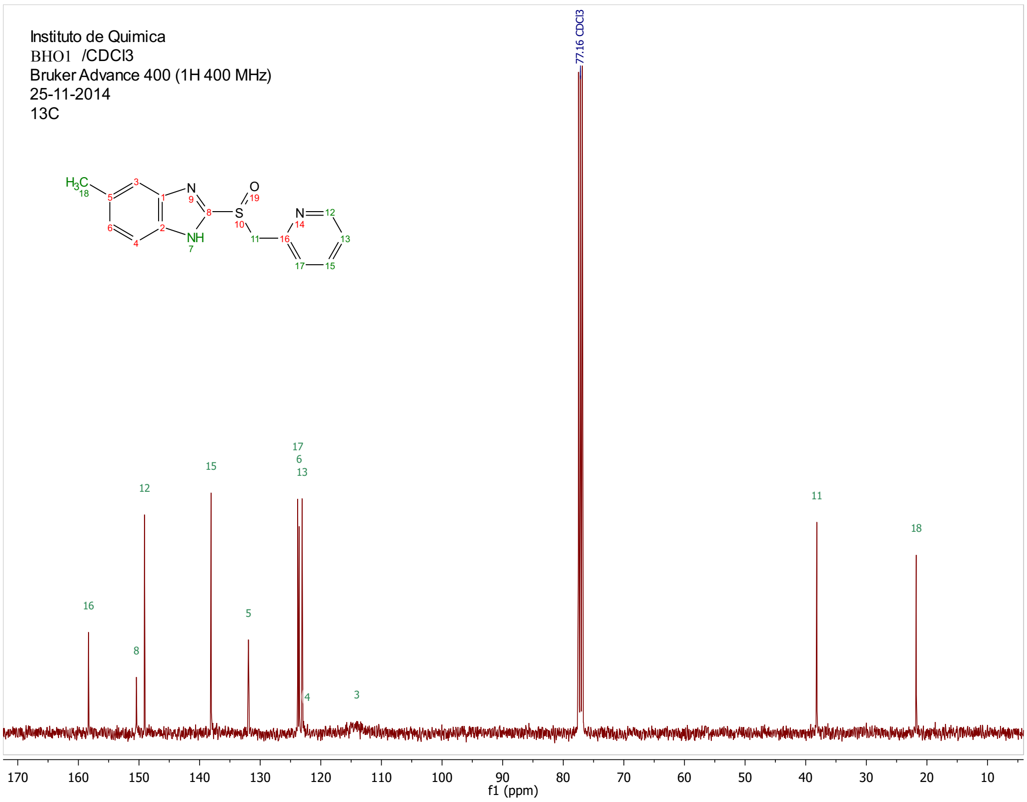
Instituto de Quimica
BHO1 /CDCl3
Bruker Advance 400 (1H 400 MHz)
25-11-2014
13C
77.16 CDCl3
f1 (ppm)

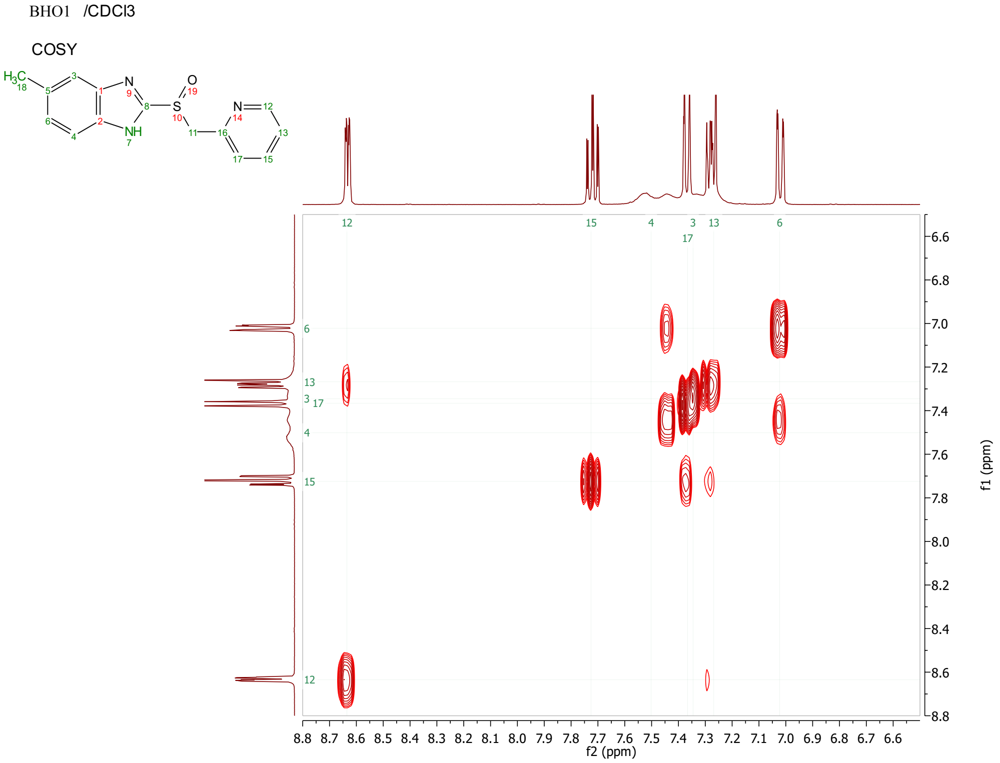
BHO1 /CDCl3
COSY
f2 (ppm)
f1 (ppm)

BHO1 /CDCl3

HSQCed

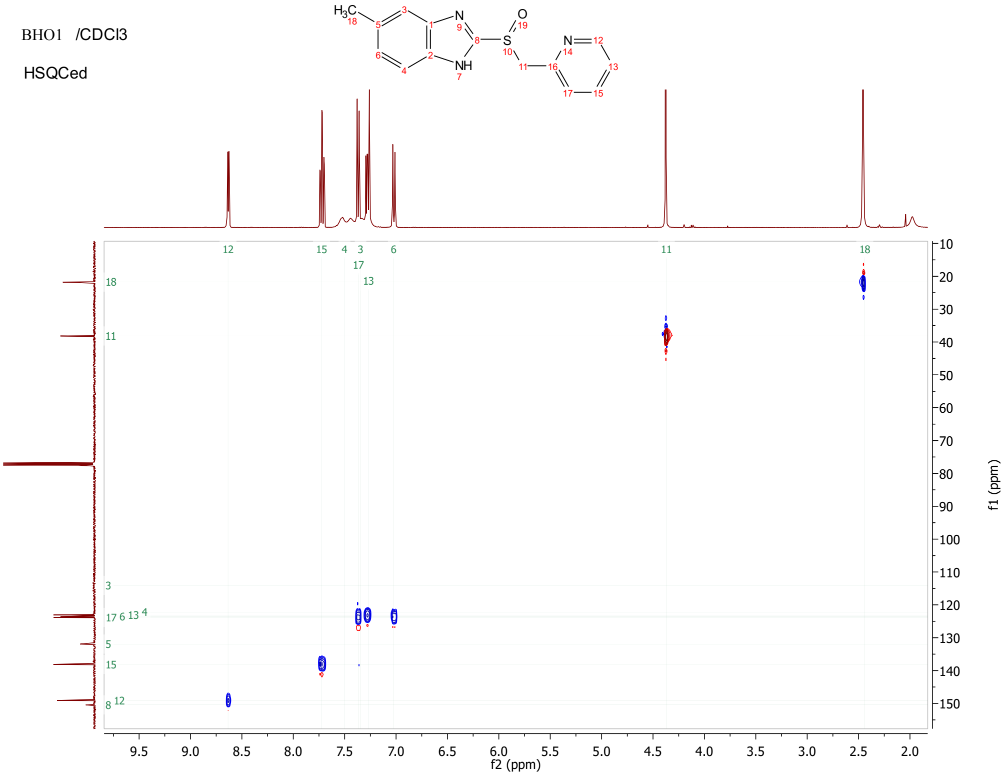

BHO1 /CDCl3

HMBC

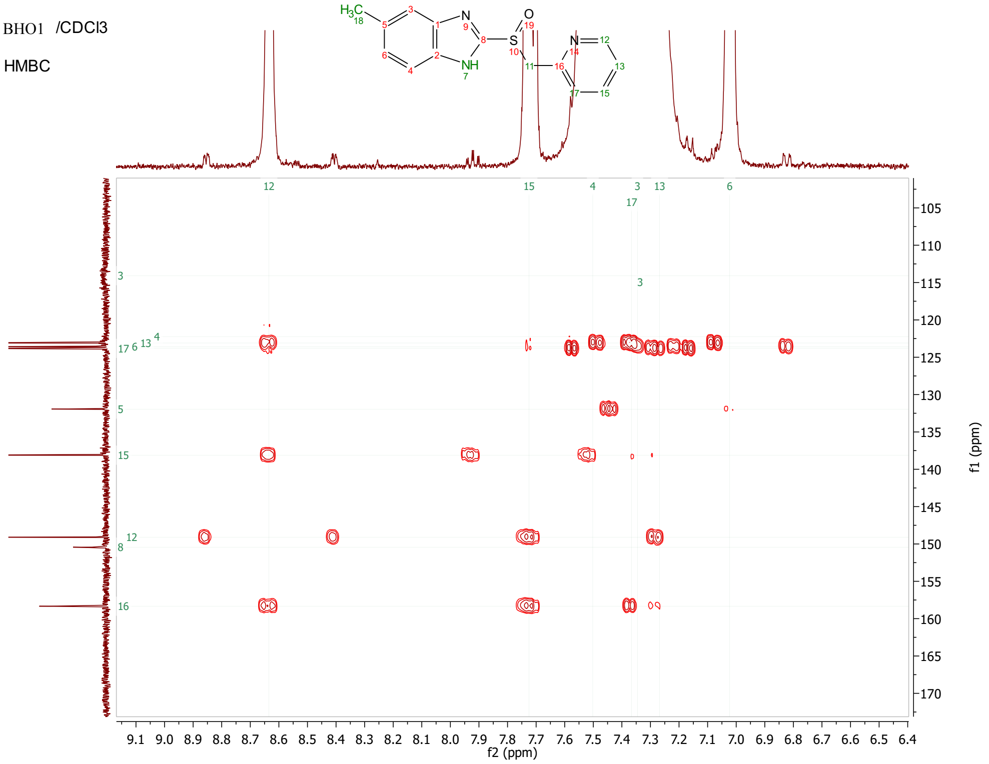

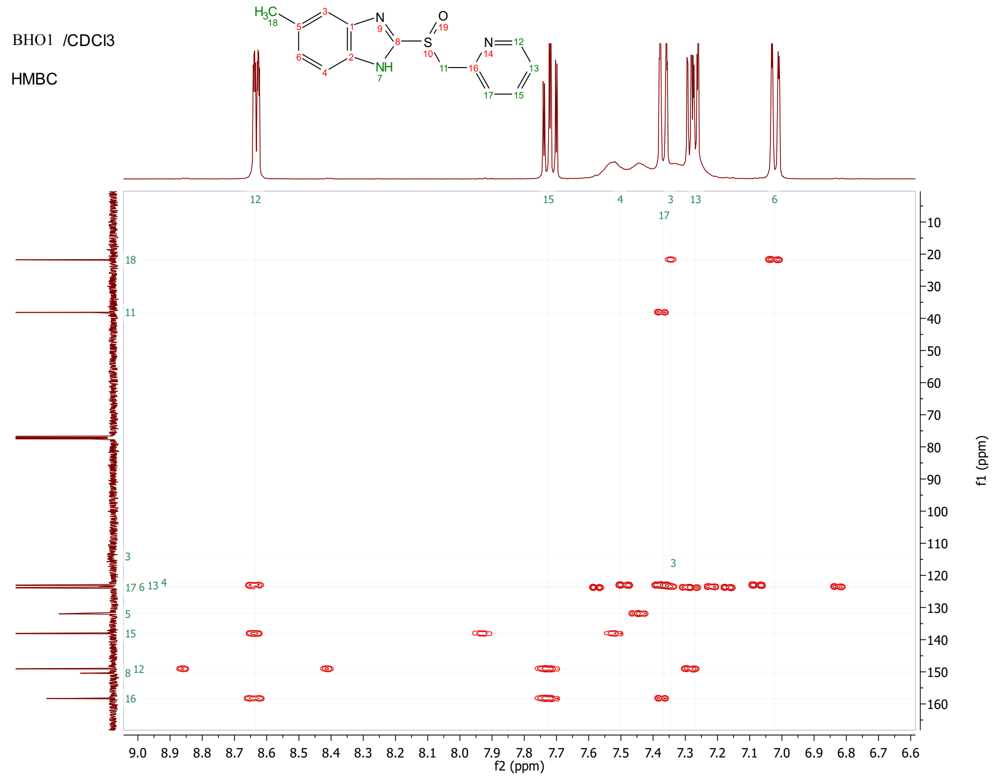
BHO1 /CDCl3
HMBC
f2 (ppm)
f1 (ppm)

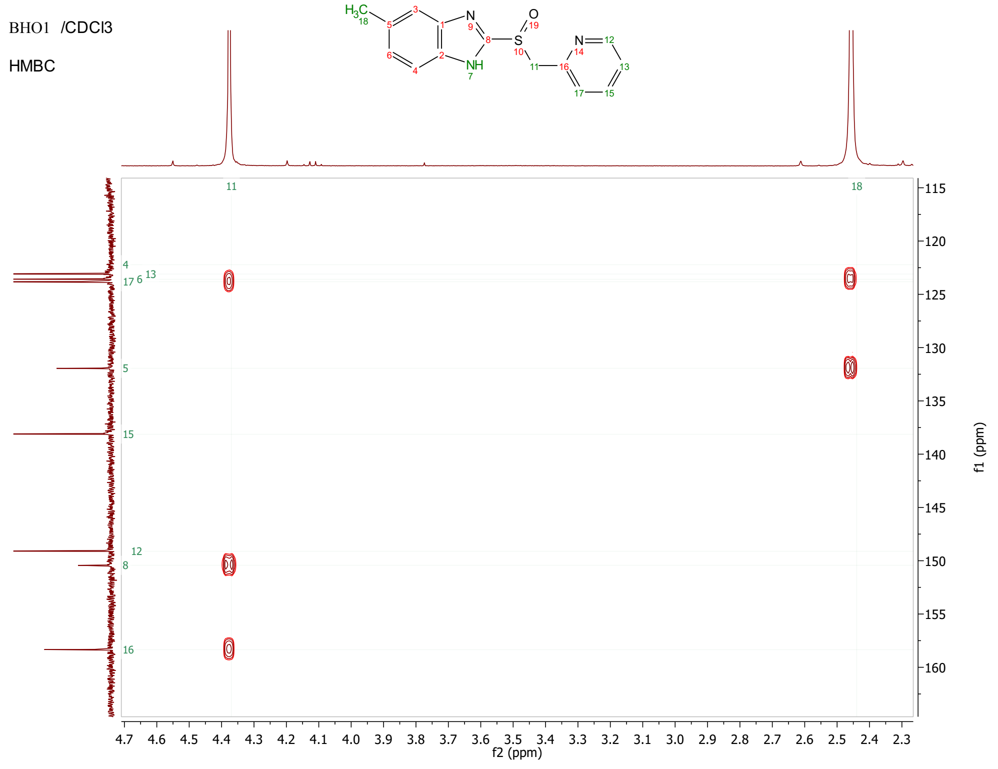
BHO1 /CDCl3
HMBC
f2 (ppm)
f1 (ppm)

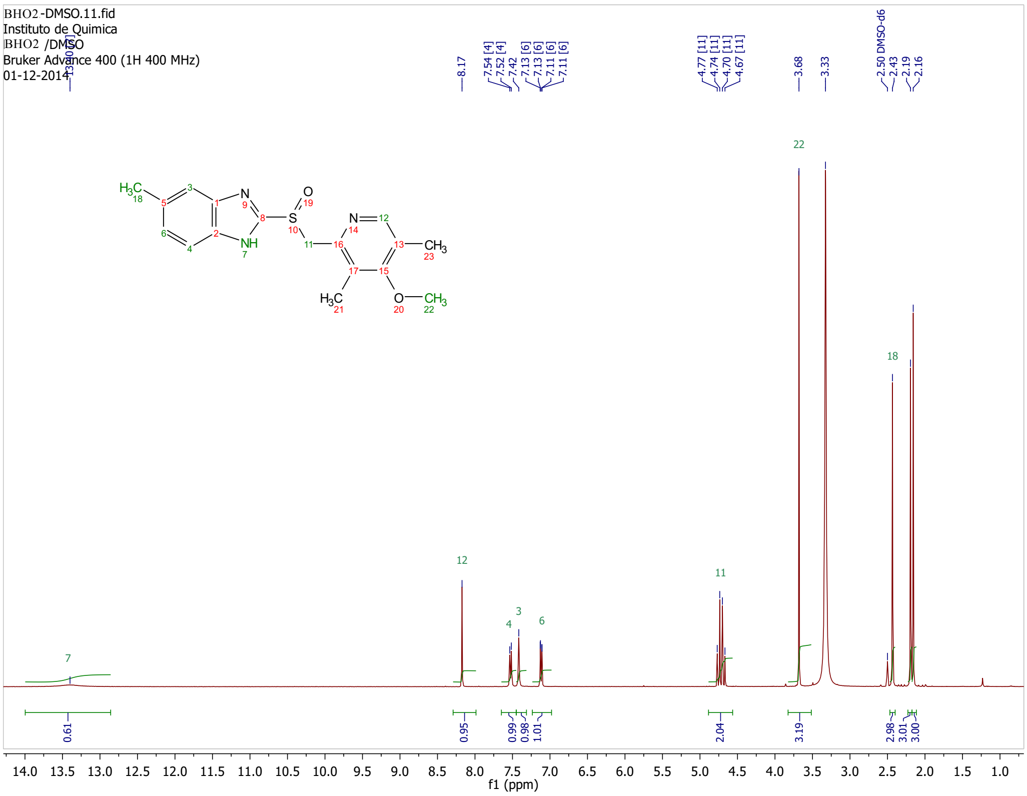
BHO2-DMSO.11.fid
Instituto de Quimica
BHO2 /DMSO
Bruker Advance 400 (1H 400 MHz)
01-12-2014
f1 (ppm)

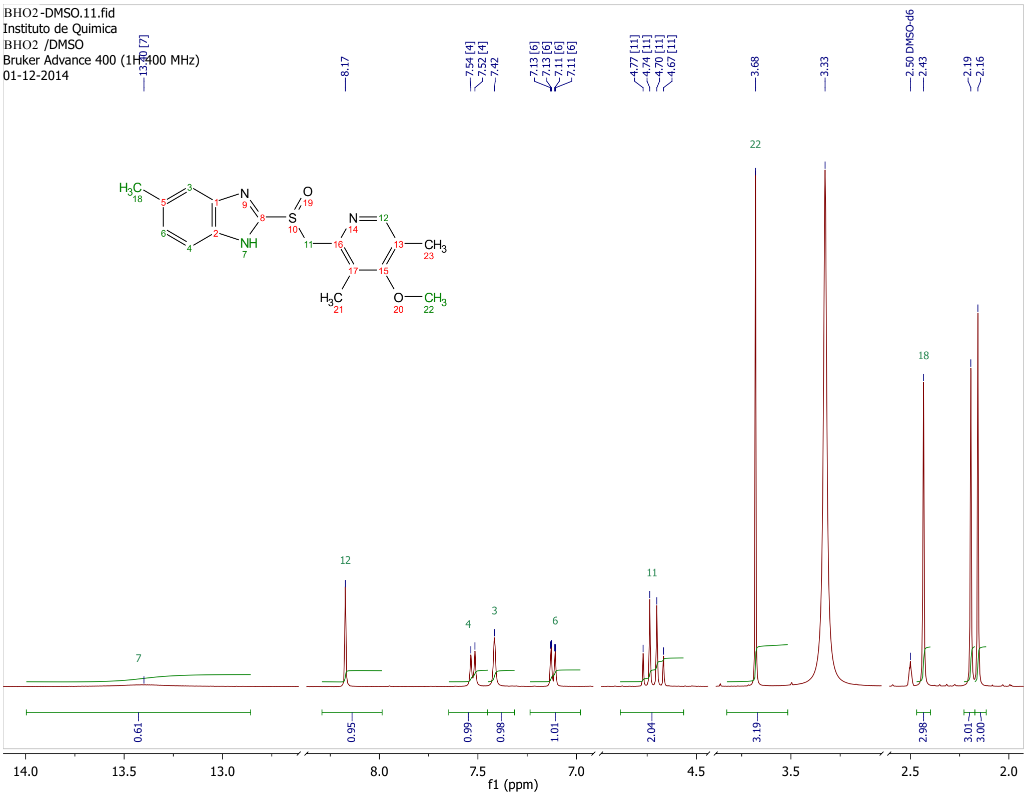
BHO2-DMSO.11.fid
Instituto de Quimica
BHO2 /DMSO
Bruker Advance 400 (1H 400 MHz)
01-12-2014
f1 (ppm)

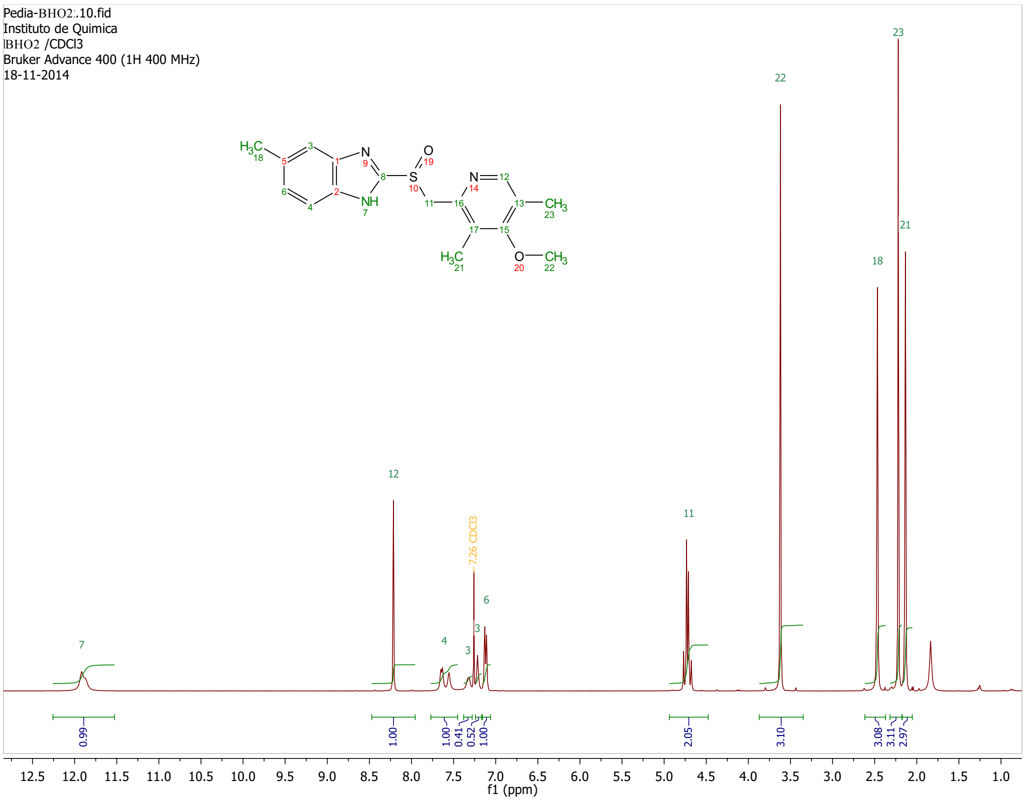
Pedia-BHO2.10.fid
Instituto de Quimica
BHO2 /CDCl3
Bruker Advance 400 (1H 400 MHz)
18-11-2014
7.26 CDCl3
f1 (ppm)

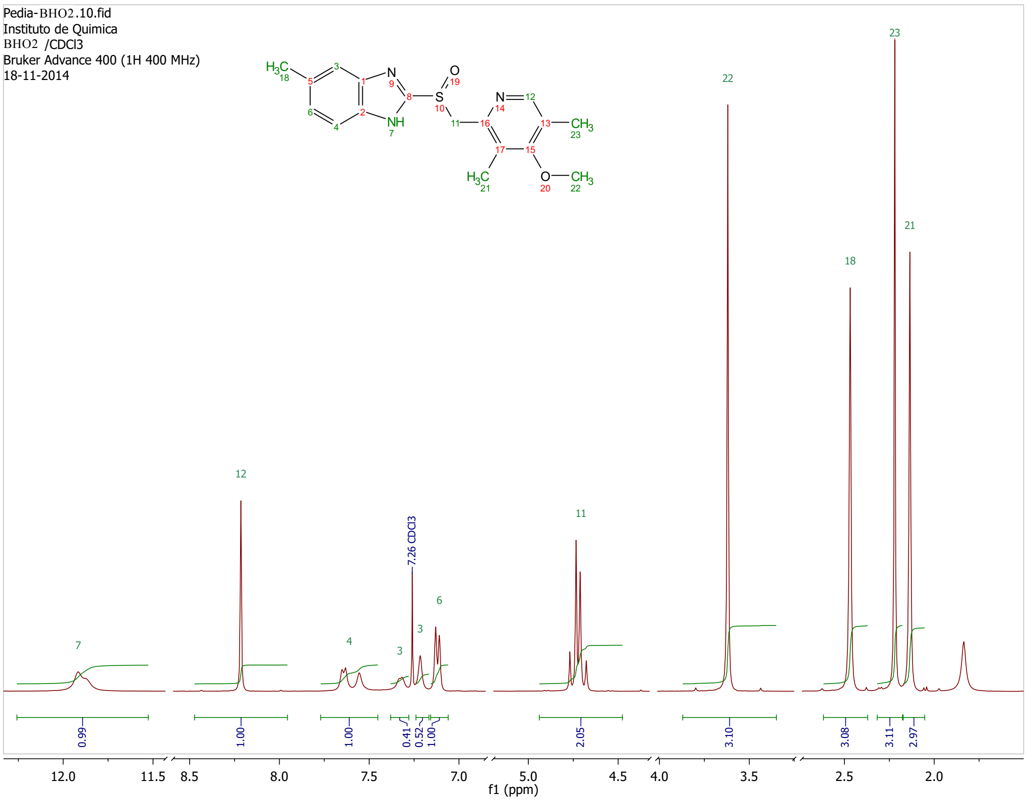
Pedia-BHO2.10.fid
Instituto de Quimica
BHO2 /CDCl3
Bruker Advance 400 (1H 400 MHz)
18-11-2014
7.26 CDCl3
f1 (ppm)

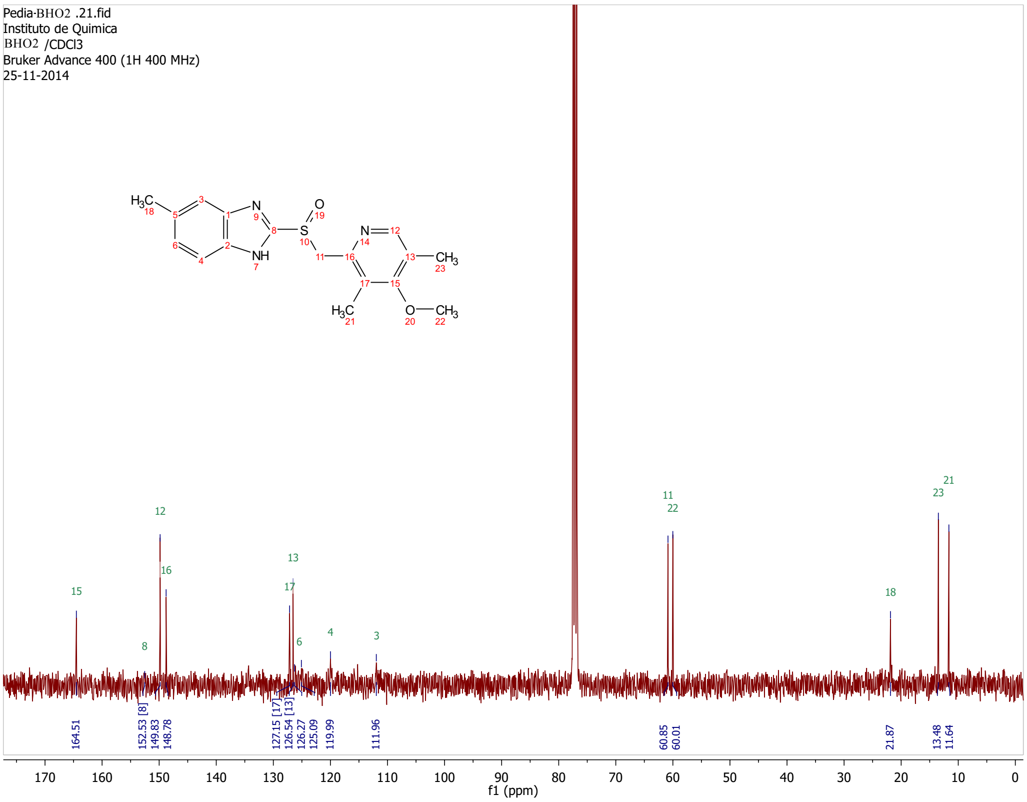
Pedia-BHO2 .21.fid
Instituto de Quimica
BHO2 /CDCl3
Bruker Advance 400 (1H 400 MHz)
25-11-2014
164.51
152.53 [8]
149.83
148.78
127.15 [17]
126.54 [13]
126.27
125.09
119.99
111.96
60.85
60.01
21.87
13.48
11.64
f1 (ppm)

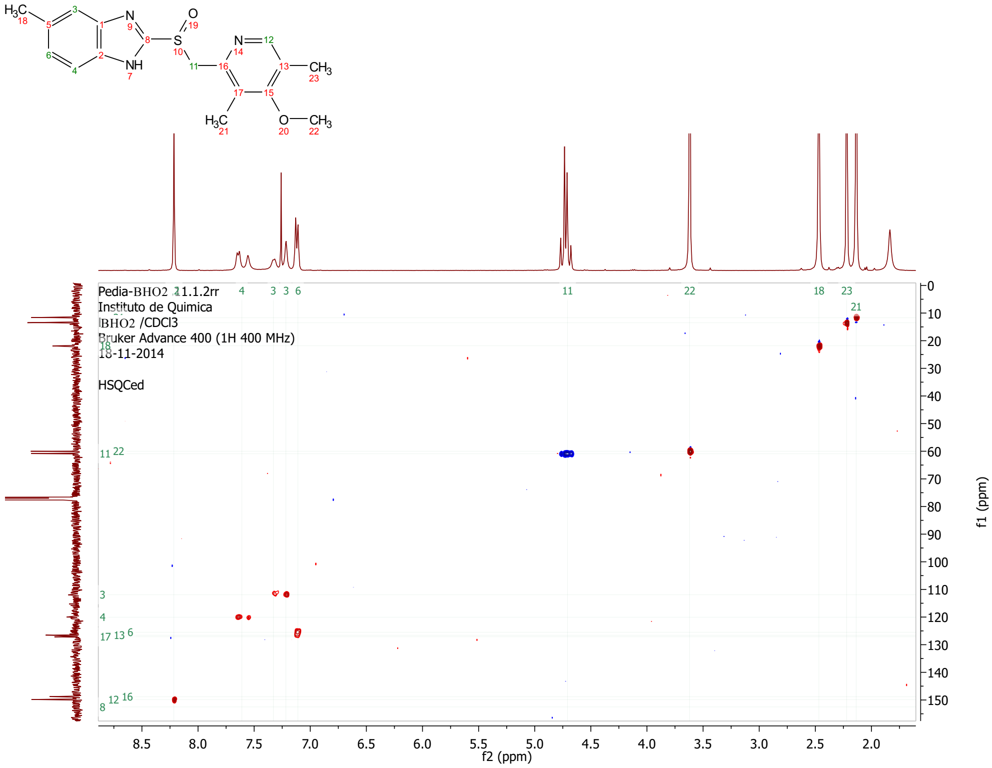
Pedia-BHO2 11.1.2rr
Instituto de Quimica
BHO2 /CDCl3
Bruker Advance 400 (1H 400 MHz)
18-11-2014
HSQCed
f2 (ppm)
f1 (ppm)

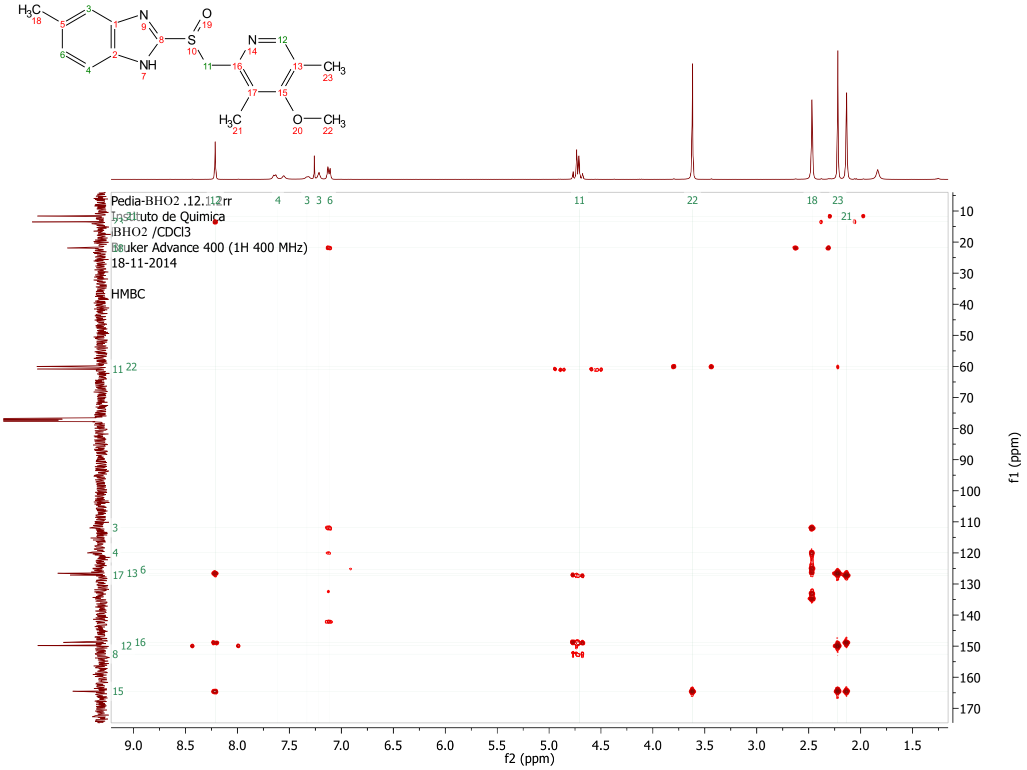
Pedia-BHO2 .12.1.2rr
Instituto de Quimica
BHO2 /CDCl3
Bruker Advance 400 (1H 400 MHz)
18-11-2014
HMBC
f2 (ppm)
f1 (ppm)

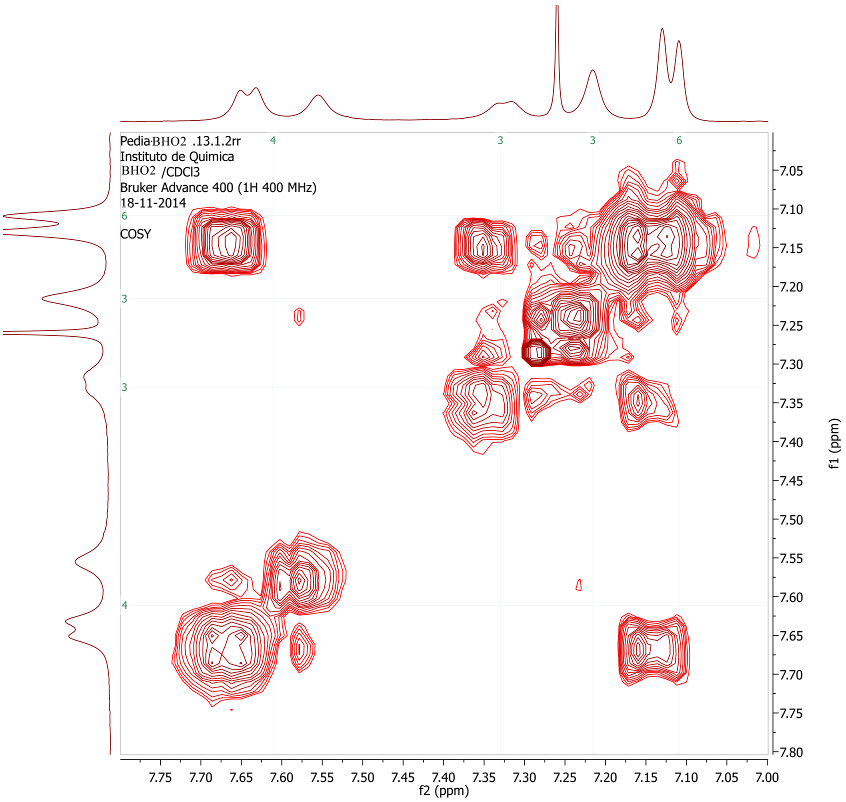
Pedia-BHO2 .13.1.2rr
Instituto de Quimica
BHO2 /CDCl3
Bruker Advance 400 (1H 400 MHz)
18-11-2014
COSY
f2 (ppm)
f1 (ppm)

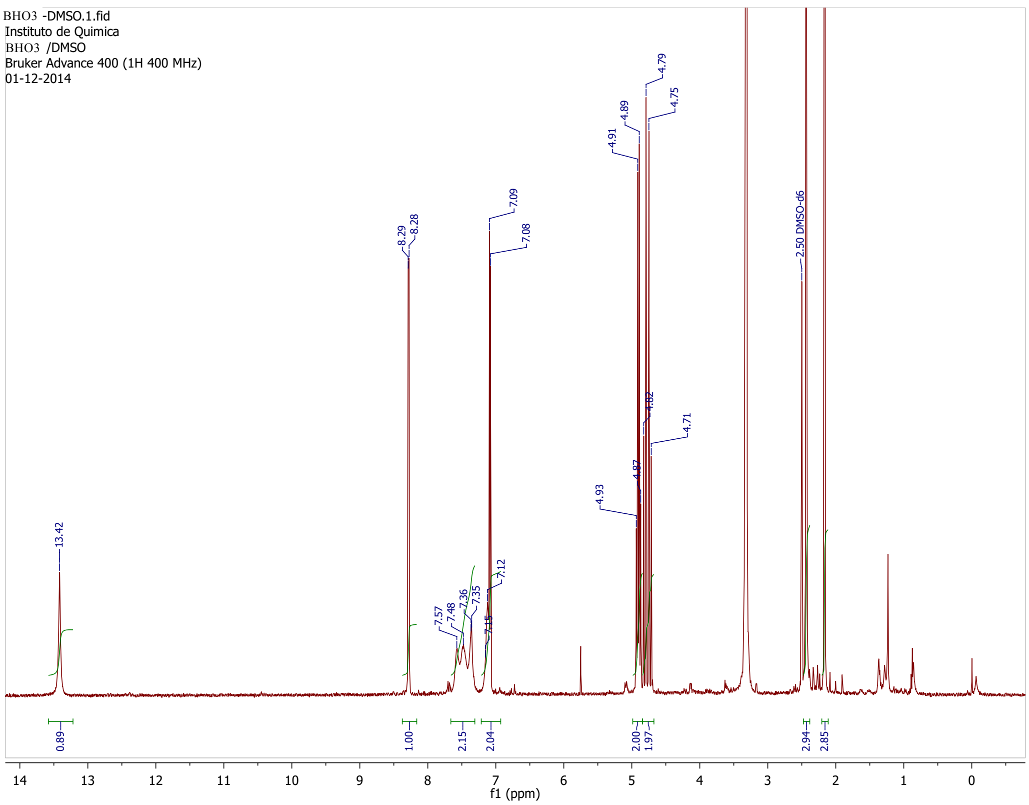
BHO3 -DMSO.1.fid
Instituto de Quimica
BHO3 /DMSO
Bruker Advance 400 (1H 400 MHz)
01-12-2014
f1 (ppm)

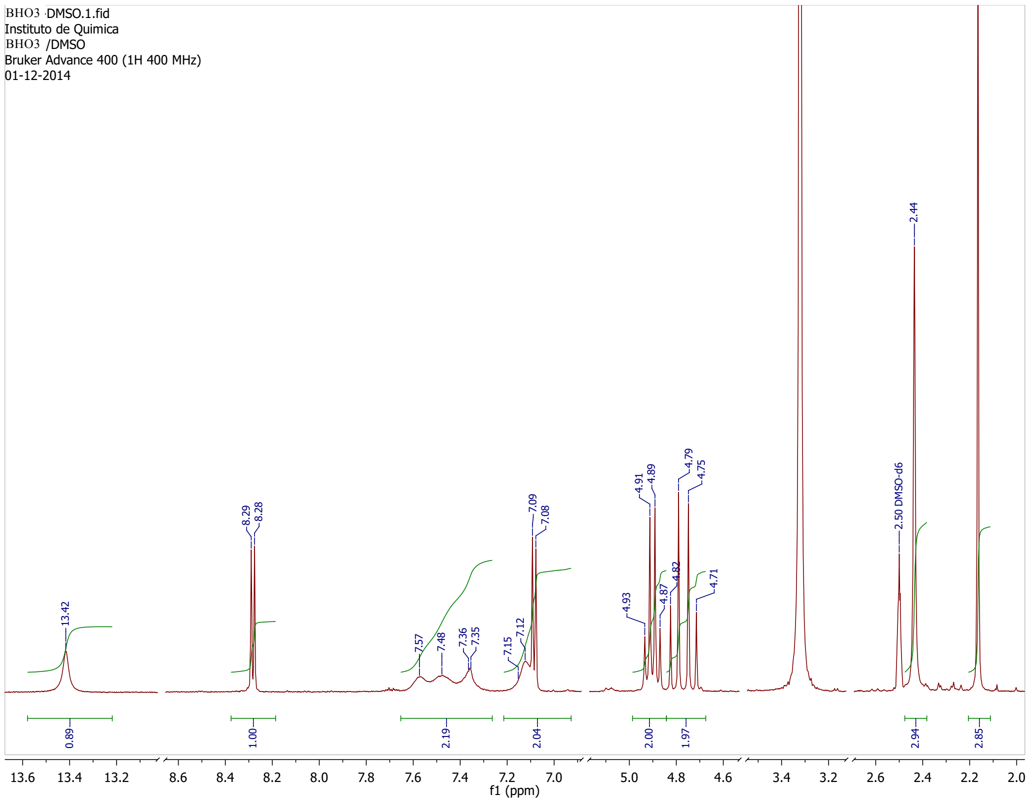
BHO3 -DMSO.1.fid
Instituto de Quimica
BHO3 /DMSO
Bruker Advance 400 (1H 400 MHz)
01-12-2014
f1 (ppm)

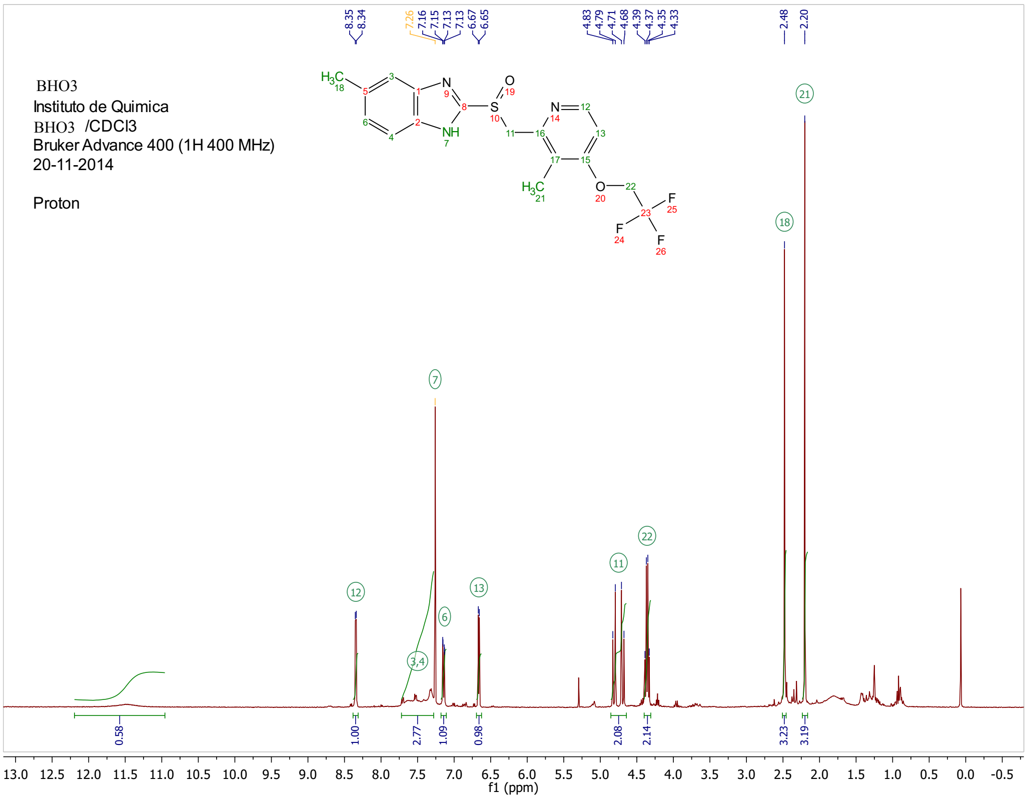

BHO3
Instituto de Quimica
BHO3 /CDCl3
Bruker Advance 400 (1H 400 MHz)
20-11-2014
Proton
f1 (ppm)

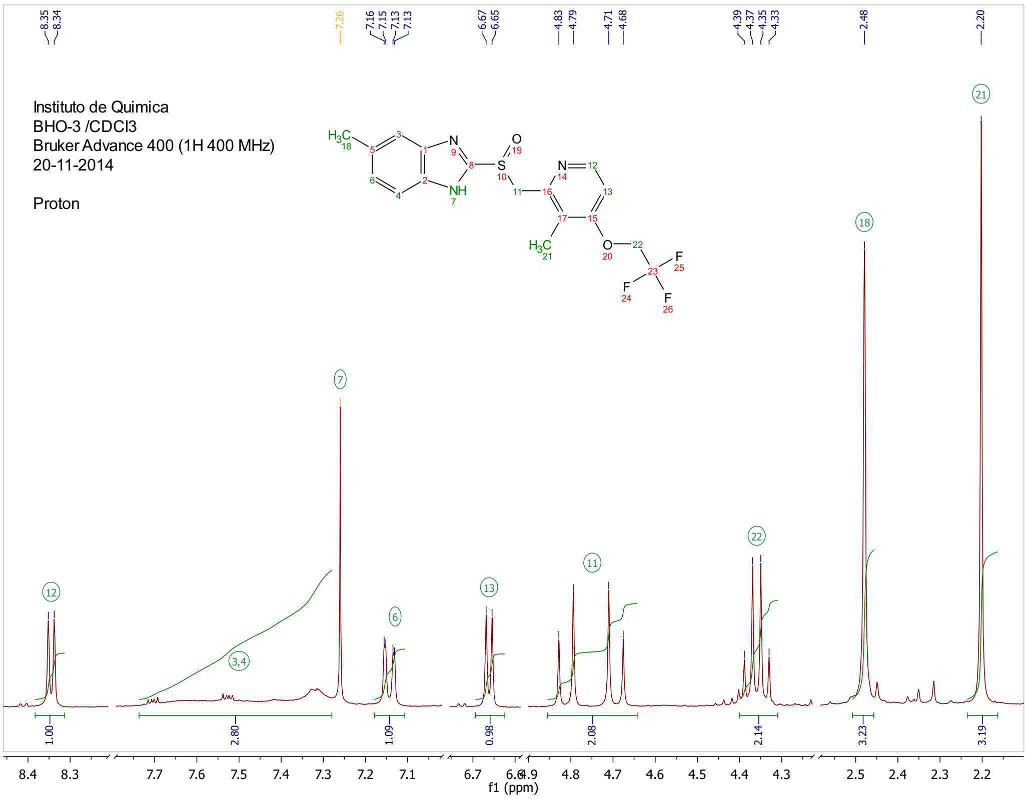

Instituto de Quimica
BHO-3 /CDCl3
Bruker Advance 400 (1H 400 MHz)
20-11-2014
Proton
f1 (ppm)

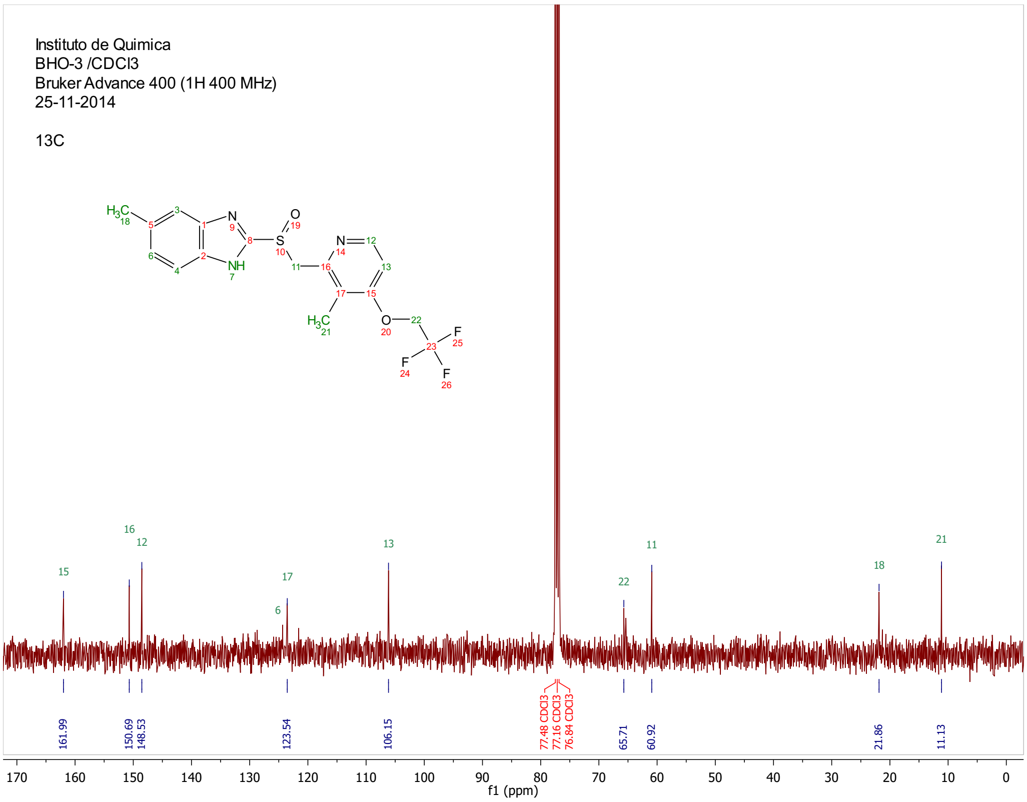

Instituto de Quimica
BHO-3 /CDCl3
Bruker Advance 400 (1H 400 MHz)
25-11-2014
13C
161.99
150.69
148.53
123.54
106.15
77.48 CDCl3
77.16 CDCl3
76.84 CDCl3
65.71
60.92
21.86
11.13
f1 (ppm)

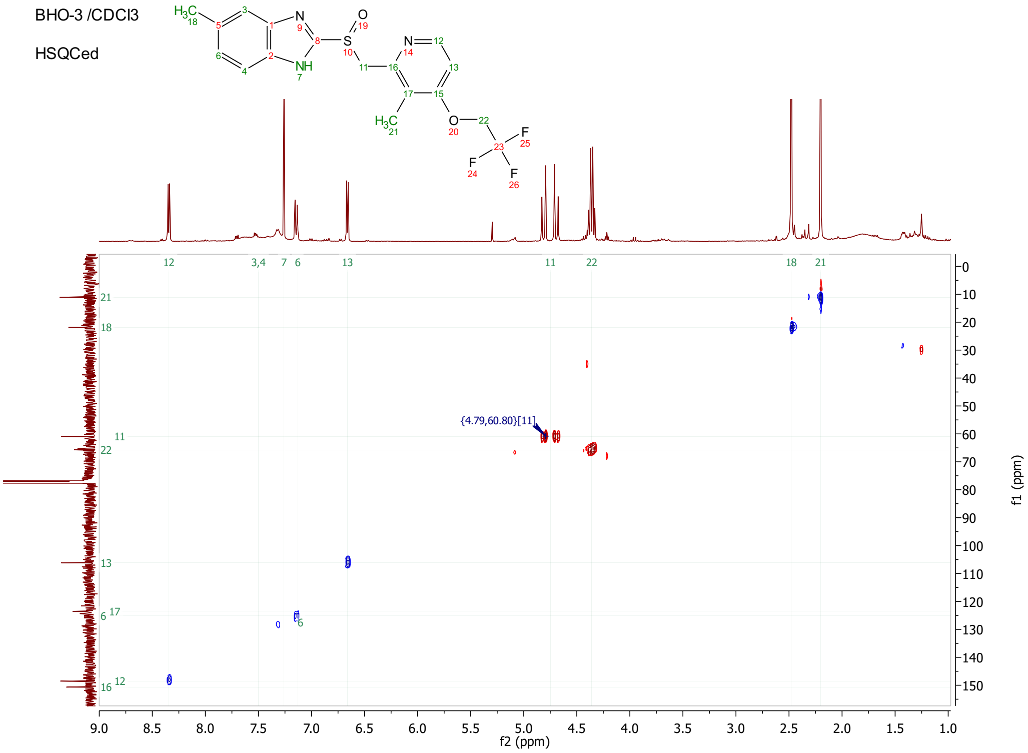

BHO-3 /CDCl3
HSQCed
{4.79,60.80}[11]
f2 (ppm)
f1 (ppm)

BHO-3 /CDCl3

HMBC

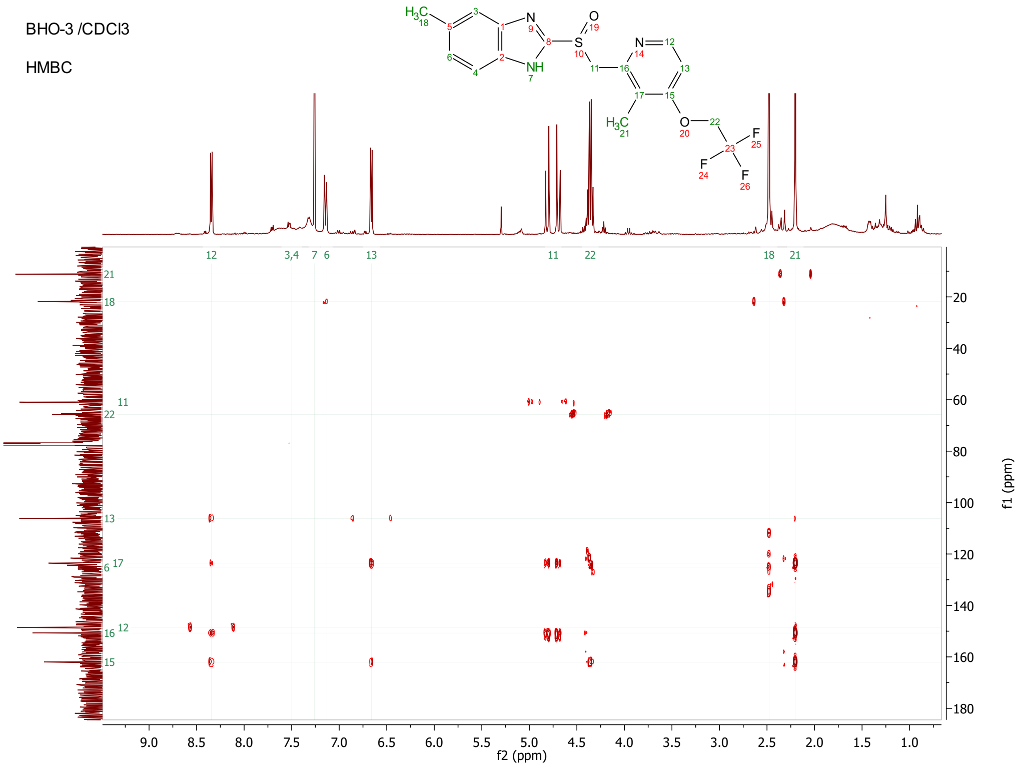

BHO-3 /CDCl3

HMBC

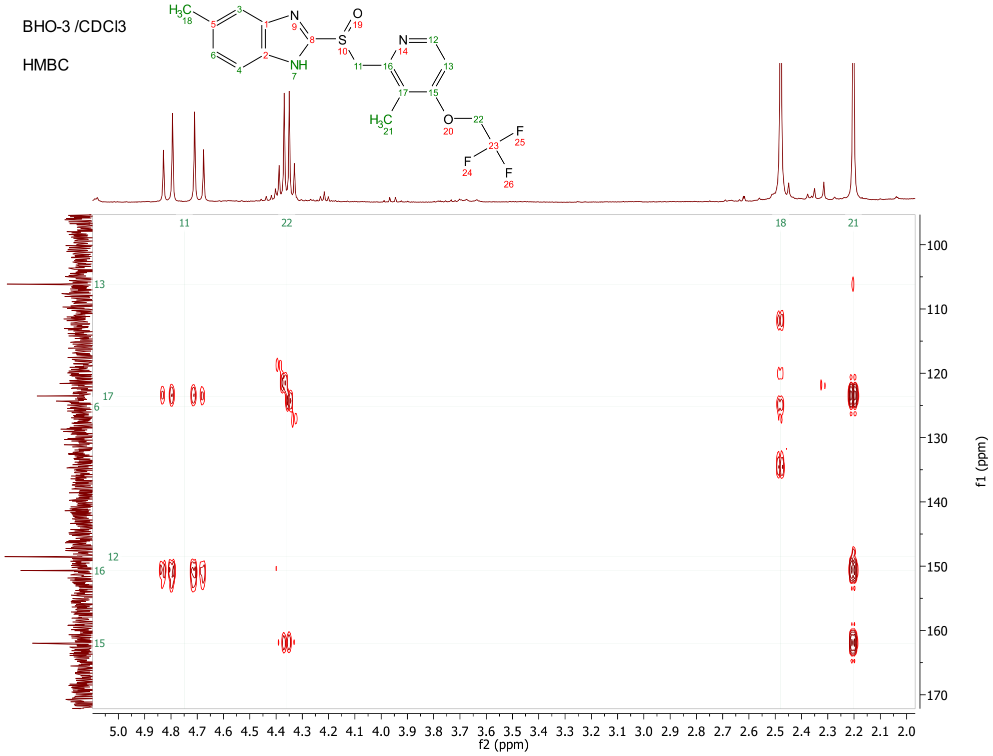

S2. Sequence Alignment between Giardia TIM (GlTIM) and Human TIM (HsTIM). The Cys residues in both enzymes are highlighted in bold plus underlined. Alignment was performed by using Clustal W2 software.

```
                 10         20         30         40         50         60
GlTIM   GHMPARRPFI GGNFKCNGSL DFIKSHVAAI AAHKIPDSVD VVIAPSAVHL STAIAANTSK
HsTIM   --APSRKFFV GGNWKMNGRK QSLGELIGTL NAAKVPADTE VVCAPPTAYI DFAR-QKLDP

                 70         80         90        100        110        120
GlTIM   QLRIAAQNVY LEGNGAWTGE TSVEMLQDMG LKHVIVGHSE RRRIMGETDE QSAKKAKRAL
HsTIM   KIAVAAQNCY KVTNGAFTGE ISPGMIKDCG ATWVVLGHSE RRHVFGESDE LIGQKVAHAL

                130        140        150        160        170        180
GlTIM   EKGMTVIFCV GETLDERKAN RTMEVNIAQL EALGKELGES KMLWKEVVIA YEPVWSIGTG
HsTIM   AEGLGVIACI GEKLDEREAG ITEKVVFEQT KV----IADN VKDWSKVVLA YEPVWAIGTG

                190        200        210        220        230        240
GlTIM   VVATPEQAEE VHVGLRKWFA EKVCAEGAQH IRIIYGGSAN GSNCEKLGQC PNIDGFLVGG
HsTIM   KTATPQQAQE VHEKLRGWLK SNVSDAVAQS TRIIYGGSVT GATCKELASQ PDVDGFLVGG

                250       259
GlTIM   ASLKPEFMTM IDILTKTRT
HsTIM   ASLKPEFVDI INAKQ----
```

S3. **Inactivation assays of GlTIM with omeprazole**, **BHO2 or BHO3**. The experimental conditions are described in the Results section, headland 4.4.3. The pseudo-first order rate constant values (k_1) for each compound were calculated by fitting the data to the exponential decay equation $A = A_o e^{-k_1 t}$, where A_o is the initial activity value. All experiments were performed by triplicate; the bars represent the standard error. The second-order rate constant values of inactivation (k_2) were obtained by plotting the $k_1$values against the concentrations of compounds.

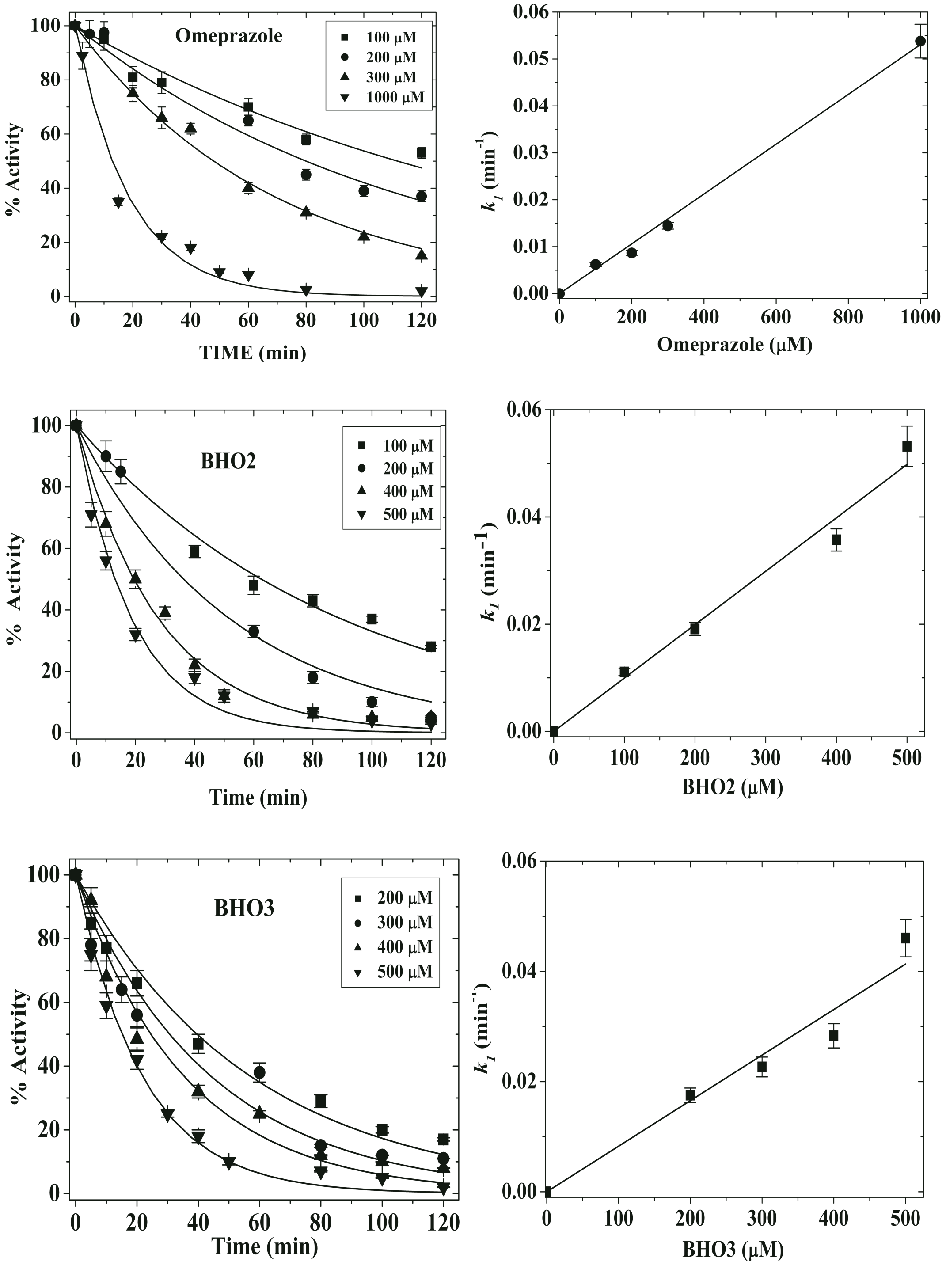

Supplement: Supplementary file 1 — Supplementary material [file 41598_2017_7612_MOESM1_ESM.pdf]
